# Cortico-pallidal beta dynamics underlie impaired turning in Parkinson’s disease

**DOI:** 10.1038/s41531-026-01421-9

**Published:** 2026-06-06

**Authors:** Poojan D. Shukla, Jessica E. Bath, Kenneth H. Louie, Hamid Fekri Azgomi, Rithvik Ramesh, Eleni Patelaki, Jacob H. Marks, Jannine P. Balakid, Doris D. Wang

**Affiliations:** 1 Department of Neurological Surgery, University of California, San Francisco, CA USA; 2 Department of Physical Therapy and Rehabilitation Science, University of California, San Francisco, CA USA

**Keywords:** Neurology, Neuroscience

## Abstract

Turning while walking is one of the most common yet complex human movements, and it is frequently impaired in Parkinson’s disease (PD), leading to falls and loss of independence. The neural mechanisms underlying this difficulty remain unclear. Using chronically implanted devices that simultaneously record and stimulate the brain, we repeatedly measured activity in the motor cortex and basal ganglia of five individuals with PD during natural walking and turning. Successful turns were marked by reduced beta-band activity and flexible communication between cortical and pallidal regions, whereas impaired turns showed excessive beta synchrony that rigidly constrained movement. Medication and deep brain stimulation improved turning through distinct circuit mechanisms, dopamine suppressing abnormal pallidal beta activity and its communication with the cortex, and stimulation restoring cortical flexibility. These findings reveal how dynamic cortical–basal ganglia interactions may enable complex movement and establish circuit targets for adaptive brain stimulation to reduce falls in PD.

## Introduction

Axial motor deficits, including impairments in gait, balance, and turning, are among the most disabling symptoms of Parkinson’s disease (PD)^1^. Because axial motor behaviors are central to daily function, difficulty performing them disproportionately reduces quality of life. Turning is a key axial behavior and a fundamental component of human gait. On average, individuals perform over 700 turns per day, and 35–45% of daily steps involve turning during routine activities^2,3^. In PD, however, turning is frequently impaired as greater than 50% of patients with PD report difficulty turning^4,5^. Compared to healthy individuals, patients with PD exhibit slower turning speeds, increased step counts, reduced turning angles and arcs, and greater variability across these metrics^3,5–10^. Moreover, turns in PD are often less stable when matched for speed with healthy subjects and are typically described as “en-bloc”, a pattern marked by impaired axial rotation that prevents segmental rotation of the head, shoulders, and pelvis in a smooth fashion^9,11^. Freezing of gait, characterized by the sudden and brief inability to step, is also more prevalent during turns^8,10,12^. Collectively, these difficulties substantially increase fall risk and contribute to the overall burden of axial disability^6,8,9,11–14^.

Pharmacological and surgical treatments for PD, including levodopa and deep-brain stimulation (DBS), have demonstrated variable and often unpredictable effects on axial symptoms such as turning. While dopamine replacement therapy can improve gait and turning metrics in some patients, such as speed and stride length, it may paradoxically exacerbate postural instability and impair balance in some individuals^1,15–18^. Similarly, axial behaviors such as balance and posture may improve, worsen, or remain unchanged following DBS^1,14,19–21^. These inconsistent therapeutic responses are further complicated by the absence of standardized tools to evaluate turning quality. For instance, the postural instability and gait difficulty (PIGD) score of the MDS-UPDRS III does not specially assess turning, and other clinical assessments of balance, such as the Mini-BEST test, offer only coarse ratings of turn performance^1,22^. As a result, existing clinical rating scales may lack the resolution necessary to capture the full spectrum of turning strategies or to detect meaningful changes across individuals^6,23^.

Despite the ubiquity and clinical relevance of turning during gait, the neurophysiological mechanisms underlying this behavior remain poorly understood. Turning requires the seamless integration of sensory inputs, motor planning, and dynamic postural adjustments, relying on coordinated activity across cortical and subcortical structures, including the basal ganglia, cerebellum, and sensorimotor cortices^24–27^. Impaired turning in PD may result from inadequate cortical compensation for basal ganglia dysfunction, but direct neurophysiological evidence is limited.

**Fig. 1:**
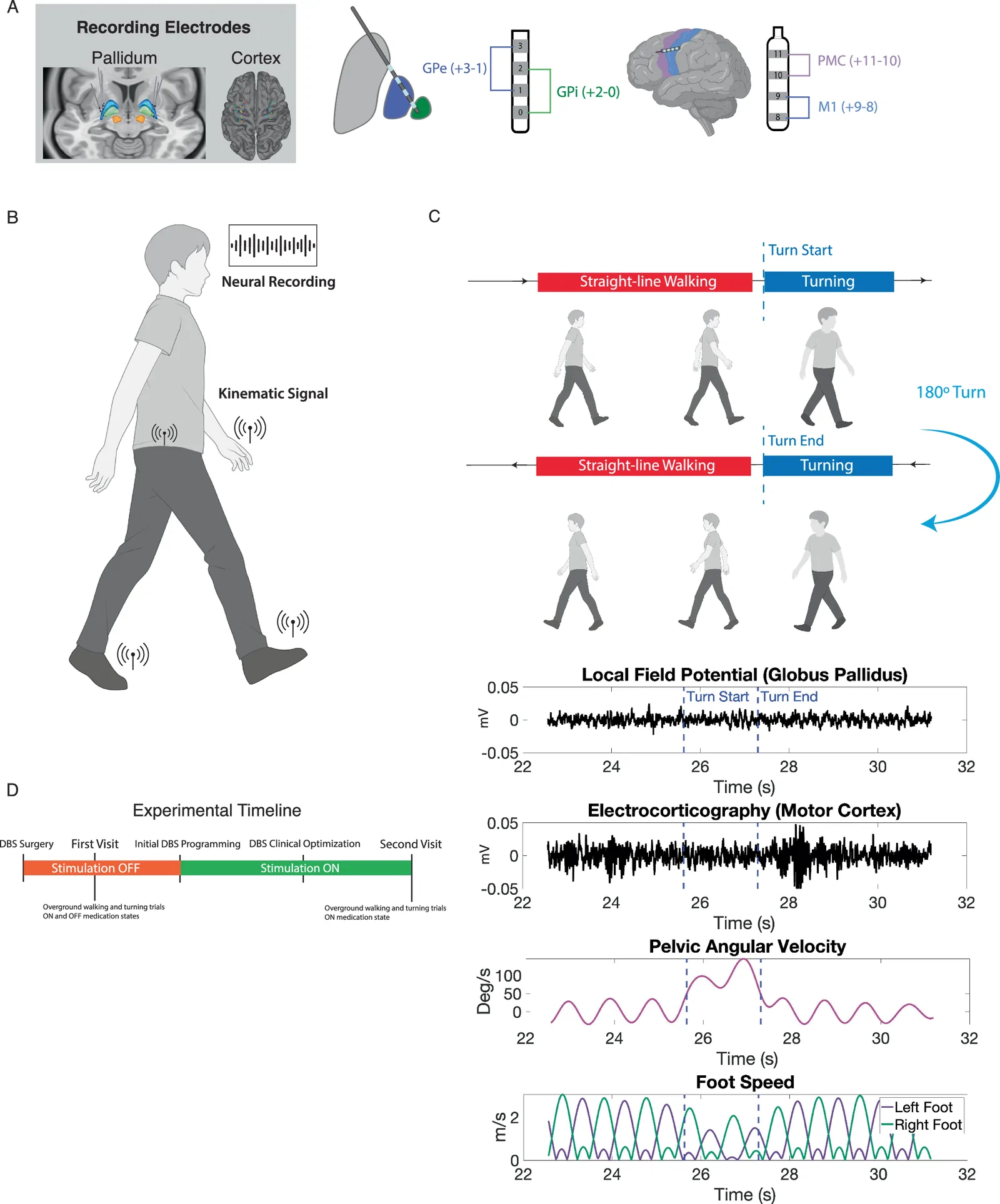
Experimental overview and workflow. **A** Implanted bidirectional neural interface facilitates neural recording from the pallidum and cortex. **B** Kinematic signals are recorded from body-worn IMU sensors during overground walking task. **C** Overview of walking task. Subjects completed trials of walking in a loop featuring 180-degree turns. The direction of turning was prespecified. Also shown are traces of pelvic angular velocity and foot speed, which are recorded from body-worn IMUs, as well as time series of local field potential and electrocorticography recordings. **D** Sample timeline of stimulation ON and OFF testing along with medication states followed by each patient.

To address these gaps, we analyzed multisite intracranial recordings from individuals with PD using an investigational bidirectional neural interface during an overground walking task that included straight-ahead walking and 180° turns (Fig. 1). We first characterized how turning-related neural signatures differ from those observed during straight walking in both the globus pallidus internus (GPi) and cortex. We then introduced and validated a novel metric of turning quality, the Turning Performance Index (TPI), to assess the heterogeneous effects of levodopa and DBS on turning behavior. Finally, we examined the neurophysiological correlates of improved versus unimproved turning across treatment conditions and trained machine learning models to classify turning periods from neural recordings and predict turn quality. These findings advance our understanding of turning impairment in PD and point to modifiable neurophysiologic mechanisms that may underlie treatment responses. This work provides a foundation for developing targeted neuromodulation strategies to improve turning and other axial motor symptoms in PD.

## Results

### Cortical beta desynchronization distinguishes turning from straight-line walking

**Fig. 2:**
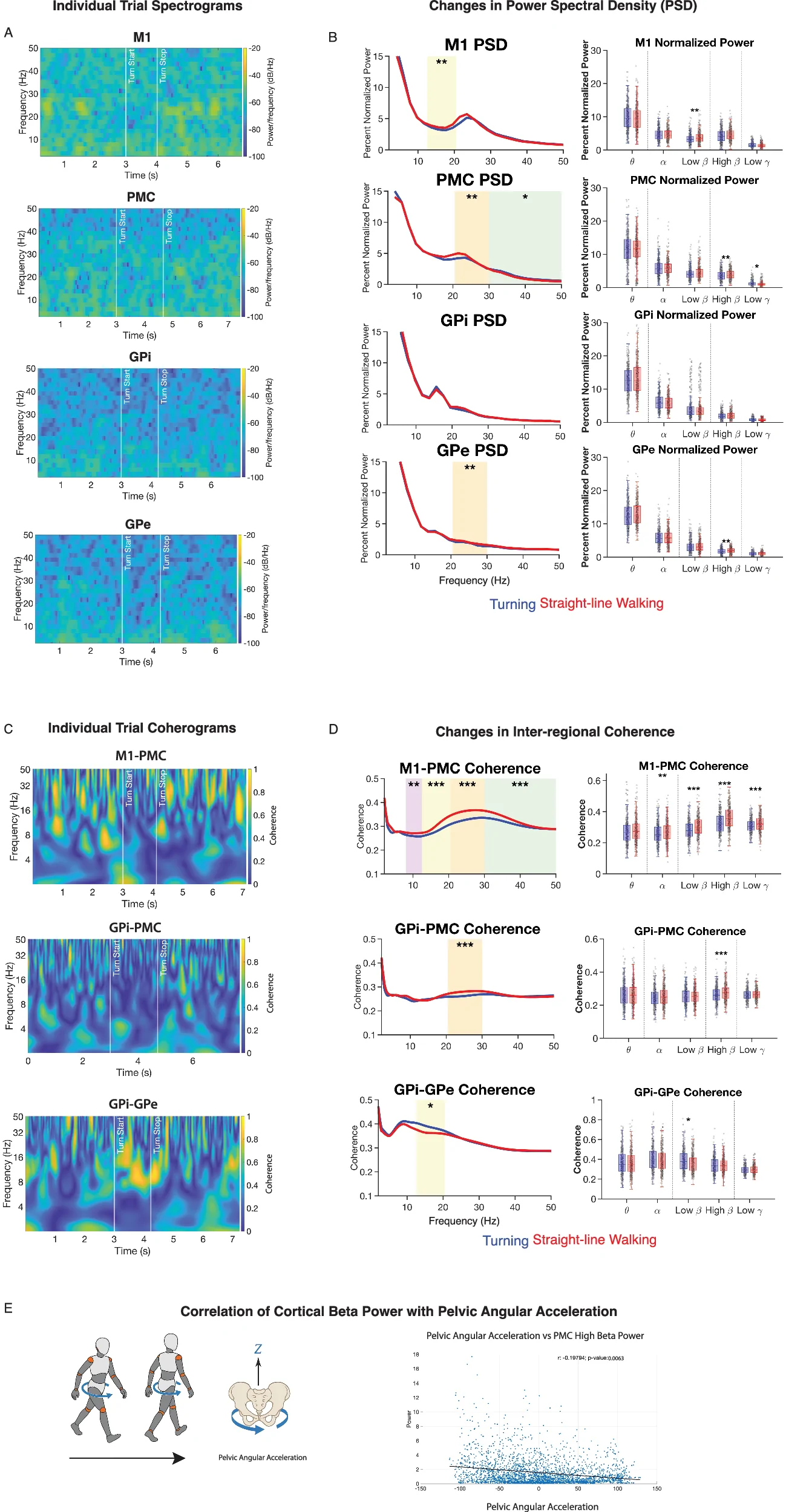
Neurophysiology of straight-line walking versus turning. **A** Spectrograms of individual turning trials and surrounding straight-line walking periods for globus pallidus internus (GPi), globus pallidus externus (GPe), primary motor (M1), and premotor (PMC) cortices. No clear differences across epochs are easily seen in the GPi while the GPe, M1, and PMC spectrograms better illustrate a decrease in beta power from “Turn Start” to “Turn Stop.” **B** Plot of normalized power spectral density averaged across all trials for straight-line walking and turning, along with box and whisker plot showing distribution of power in canonical frequency bands. All data comes from DBS OFF trials, with channels for the GPi, GPe, M1, and PMC shown. **C** Coherograms of individual turning trials and surrounding straight-line walking periods for GPi-GPe, GPi-PMC, and M1-PMC coherence. **D** Inter-regional coherence during straight-line walking and turning, along with corresponding box and whisker plots are shown for the GPi-GPe, GPi-PMC, and M1-PMC. **E** Schematic illustration of pelvic angular acceleration during turning and correlation between M1 high beta power and pelvic angular acceleration for one subject. For all panels, * indicates *p* < 0.05, ** indicates *p* < 0.01, and *** indicates *p* < 0.001. Shaded colored regions in spectral power and coherence plots indicate a significant difference in the specified frequency band (theta 4–8 Hz, alpha 8.5–12 Hz, low beta 13–20 Hz, high beta 20.5–30 Hz, and low gamma 30.5–50 Hz).

We first explored differences in neural biomarkers between turning and straight-line overground walking epochs in both ON and OFF med states, prior to DBS activation. Across five subjects and eight implanted hemispheres, we analyzed 394 turning trials with 399 straight-line walking epochs. Compared to straight-line walking (Str), both M1 and PMC exhibited decreased normalized low beta (turning vs. Str: 3.37% vs. 3.77%, *p* = 0.0028) and high beta power (turning vs. Str: 3.64% vs. 3.93%, *p* = 0.0087) during turning (Fig. 2A, B). GPe also exhibited diminished high beta power during turning compared to straight-line walking (turning vs Str: 1.77% vs. 1.97%, *p* = 0.0061). No significant spectral differences were seen in the GPi. Subject-specific changes in power and coherence are shown in Supplementary Fig. 1.

To explore pallidal-cortical network changes associated with turning, we examined inter-regional coherence differences between turning and straight-line walking. Compared to straight-line walking, GPi-PMC (turning vs Str: 0.26 vs. 0.28, *p* = 0.0003) and M1-PMC (turning vs Str: 0.33 vs. 0.36, *p* = 1.51e-10) showed significant decrease in high beta coherence during turning (Fig. 2 C, D). There were also significant decreases in alpha (0.26 vs. 0.27, *p* = 0.0055), low beta (0.28 vs. 0.31, *p* = 1.64E-7), and low gamma (0.31 vs. 0.32, *p* = 0.00014) coherence in M1-PMC during turns. Interestingly, GPi-GPe coherence in low beta was increased during turning compared to straight-line walking (0.36 vs. 0.39, *p* = 0.018). These findings were supported by linear mixed-effects modeling as well (Supplementary Tables 3 and 4).

Overall, these spectral changes highlighted the association of decreased cortical, both M1 and PMC, beta band power during turning. This motivated us to explore if any kinematic variables were correlated with these neurophysiological differences. We found that across all five subjects, pelvic angular acceleration during turning was negatively correlated with M1 low or high beta power (correlation coefficient range: −0.04 to −0.17, Supplementary Fig. 2). PMC high beta power was also negatively correlated with pelvic angular acceleration (correlation coefficient range: −0.05 to −0.2, Supplementary Fig. 2, Fig. 2E). After correcting for temporal autocorrelation using AR(1) mixed-effects models, the M1 association reached significance in subject 1 (low beta *p* = 0.050, high beta *p* = 0.050) with subject 5 showing a trend in high beta (*p* = 0.075). The PMC high beta association reached significance in subjects 2 (*p* = 0.006) and 3 (*p* = 0.015), with subject 5 showing a trend (*p* = 0.079).

### Turning performance index quantifies interindividual improvement with medication and DBS

**Fig. 3:**
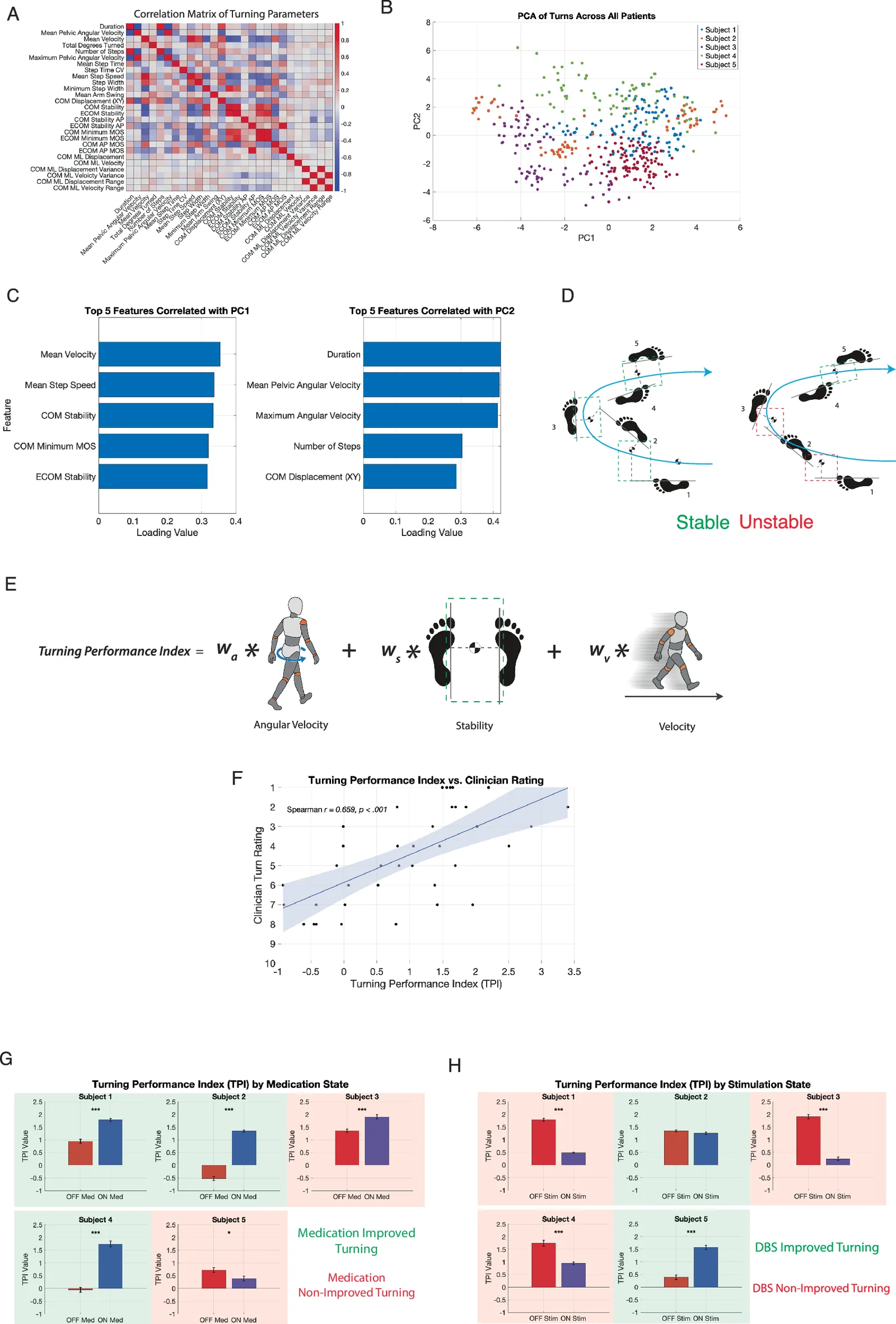
Definition and validation of a turning performance index. **A** Correlation matrix showing the correlation between all turning parameters analyzed. **B** Principal component analysis of all turns analyzed based on their turning parameters. **C** Top five turning parameters most correlated with the first and second principal component by absolute value of loading coefficient. **D** Illustration of the stability measurement. Checkered circle indicates center of mass, dashed gray lines indicate distance from center of mass to lateral edges of base of support. Green boxes indicate instances of the center of mass staying within the base of support while red boxes denote periods when the center of mass lies outside, resulting in instability. **E** Schematic illustrating the final turning performance index formulation. **F** Validation of our turning performance index with clinician rating. **G** Turning performance index applied to all subjects in stimulation off trials. Green boxes highlight subjects considered to have improved turns with medication while red boxes indicate medication non-improved. **H** DBS improved turning highlighted with green boxes and non-improved turning highlighted with red boxes. * indicates *p* < 0.05, ** indicates *p* < 0.01, and *** indicates *p* < 0.001.

To characterize turn quality, we identified 27 distinct measurements associated with turning performance based on literature review (Fig. 3A)^5–11,13,28–30^. Principal component analyses of the 461 turning trials in all subjects across all medication and stimulation states were used to identify the most salient features (Fig. 3B). The first ten principal components explained 88.23% of the variance, and the first two explained 41.4%. Analysis of the turning parameters most correlated with the first two principal components revealed that measures of turning speed, both linear and angular speed, as well as stability were most influential (Fig. 3C). Stability was assessed by measuring the position of the COM relative to the base of support, with an illustrative example in Fig. 3D showing a turn with stable steps throughout alongside a turn featuring some unstable moments. Angular velocity, stability, and linear speed were then combined into a turning performance index (Fig. 3E). Our TPI was correlated with clinical rating of turn quality (*r* = 0.659, *p* < 0.01, Fig. 3F).

To evaluate the effects of medication state or DBS on turn quality, we used our validated TPI to quantitively rate all the turns across different medication and stimulation conditions. Subjects 1–4 had significantly improved turning (all *p* < 0.001) ON med compared to OFF med with subject 3 showing the smallest change in TPI comparing ON vs OFF med conditions (1.36 vs. 1.9) among patients with medication improved turning. Subject 5 showed worse turning ON med compared to OFF med (TPI: 0.72 vs. 0.39, *p* = 0.016, Fig. 3G). Next, we evaluated the effect of DBS on turn quality. Compared to turn trials with DBS OFF, turning performance worsened for subjects 1, 3, and 4 (*p* < 0.001) with DBS ON. Turning performance did not significantly differ for subject 2 (TPI: 1.34 vs. 1.25, *p* = 0.099). Only subject 5 showed a significant improvement in turning with DBS and medication compared to medication alone (TPI: 0.39 vs. 1.57, *p* < 0.001, Fig. 3H).

### Beta band dynamics are associated with turning performance

Given the variable responses to medication and DBS, we hypothesized that turn quality is correlated with shared neurophysiological features. The three patients with the greatest response to medication (subjects 1, 2, 4) were designated as the medication responders while the subject 3 with the least response to medication and subject 5 with worse turning after medication were placed in the medication non-responder group. Although no freezing of gait episodes were observed during trials, four out of five subjects endorsed freezing of gait in their clinical history at the time of data collection (Supplementary Table 9). Because responder status did not map to freezing of gait status and given the sample size of this study, we were unable to explicitly conclude on the influence of freezing of gait on neural dynamics.

**Fig. 4:**
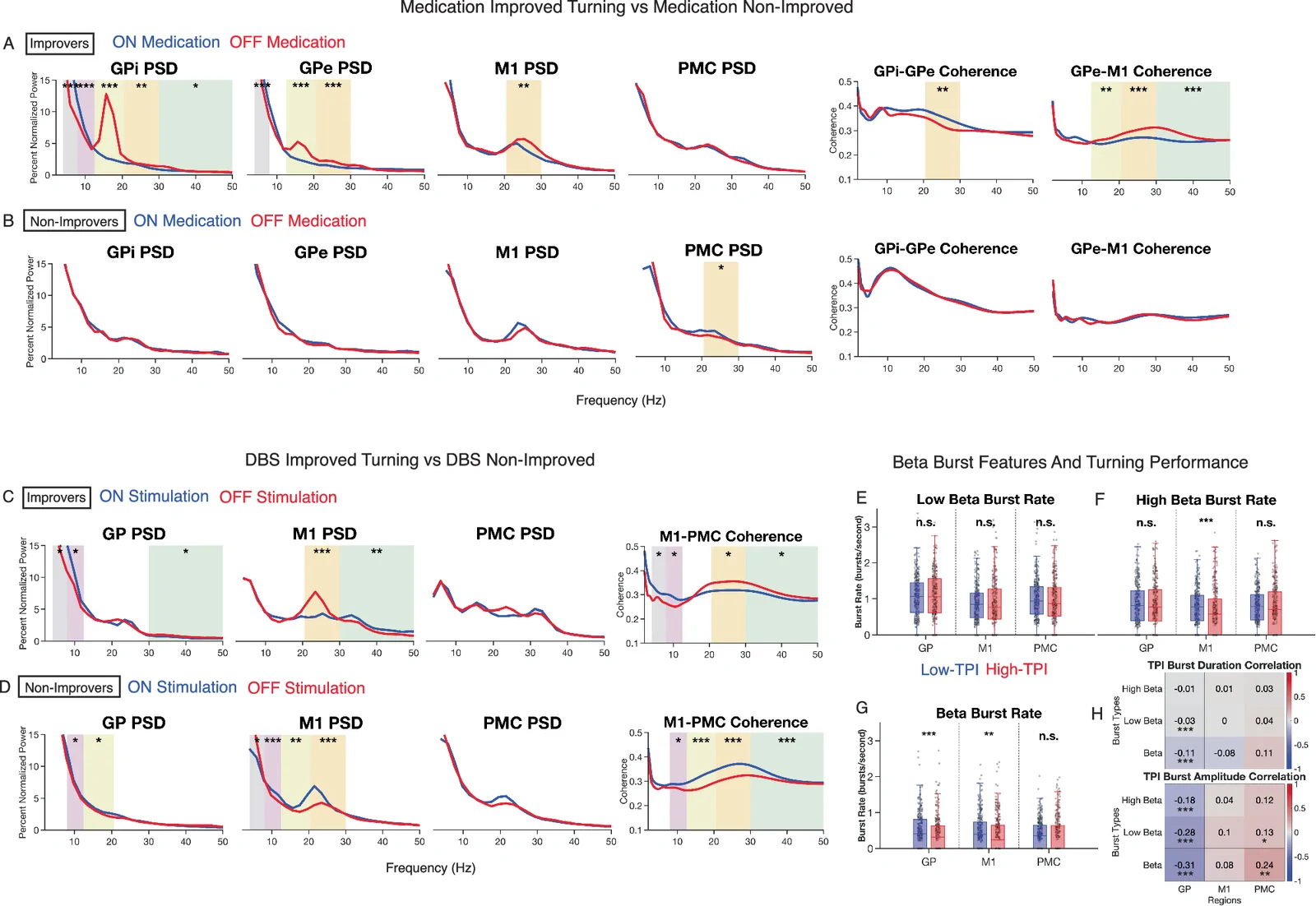
Neural features of medication and stimulation improved turns. **A** Normalized power spectral density of turning trials in the OFF medication state and ON medication state. First row represents subjects with medication improved turning and **B** second row represents non-improved turning. Globus pallidus internus (GPi), globus pallidus externus (GPe), primary motor cortex (M1), and premotor cortex (PMC) power spectral densities (PSDs) are shown. Inter-regional coherence between medication states in GPi-GPe and M1-PMC are also included. **C** Globus pallidus (GP), M1, and PMC PSD curves comparing stimulation ON and medication ON to medication ON alone among subjects with DBS improved turns in the first row and DBS non-improved turns in the (**D**) second row. M1-PMC coherence by stimulation state among these groups. **E** Average rate of low beta, **F** high beta, and **G** beta bursts during turns across brain regions in “Low TPI” and “High TPI” trials. **H** Beta burst duration and amplitude correlated with TPI across all trials. For all panels, * indicates *p* < 0.05, ** indicates *p* < 0.01, and *** indicates *p* < 0.001. Shaded colored regions in spectral power and coherence plots indicate a significant difference in the specified frequency band (theta 4–8 Hz, alpha 8.5–12 Hz, low beta 13–20 Hz, high beta 20.5–30 Hz, and low gamma 30.5–50 Hz).

In medication responders, turning in the ON state was characterized by broad suppression of beta power in both the pallidum and cortex, accompanied by reduced cortico-pallidal coherence. By contrast, non-responders showed little change across states. In the GPi, low beta (7.79% to 2.64%, *p* = 6.71E-13) and high beta power (1.80% to 1.43%, *p* = 0.0035; Fig. 4A, B) decreased substantially with medication. A similar pattern was seen in the GPe, where low beta power decreased from 4.16% to 2.33% (*p* = 5.63E-9) and high beta from 1.98 to 1.35% (*p* = 3.44E-9; Fig. 4B) in the ON state. Cortical changes were most prominent in M1, where high beta power decreased in responders (4.99–4.02%, *p* = 0.0017), and PMC high beta power increased in non-responders (2.83–3.28%, *p* = 0.048). Connectivity analyses revealed that in the medication responders, GPe–M1 coherence decreased across low beta (0.27–0.25, *p* = 0.0028), high beta (0.30–0.27, *p* = 1.4E-7), and low gamma bands (0.28–0.26, *p* = 2.88E-6) ON med, while GPi–GPe coherence increased ON med (0.32–0.36, *p* = 0.0082). Individual subject responses to medication are summarized in Supplementary Fig. 3.

To examine the neurophysiological changes associated with DBS stimulation, subjects 2 and 5 were classified as responders, as their turning performance improved or did not significantly worsen with DBS, while subjects 1, 3, and 4 were considered non-responders due to worsened turning performance. DBS responders showed cortical beta suppression and reduced cortico-cortical coherence, whereas non-responders frequently exhibited the opposite pattern. In responders, M1 high beta power decreased significantly with stimulation (5.65–3.83%, *p* = 1.95E-7), accompanied by an increase in low gamma power (1.86–2.44%, *p* = 0.0062). In contrast, DBS non-responders demonstrated increases in M1 low beta (3.22–4.24%, *p* = 0.0031) and high beta power (3.61–4.50%, *p* = 3.91E-5; Fig. 4C, D) ON DBS compared to OFF DBS. Pallidal beta power changes were only seen in non-responders, where GP low beta power increased during stimulation (2.88–3.27%, *p* = 0.045). Connectivity analyses further highlighted these divergent patterns: in responders, DBS decreased M1–PMC coherence in high beta (0.35–0.32, *p* = 0.021) and low gamma bands (0.31–0.29, *p* = 0.021), while non-responders showed marked increases in low beta (0.28–0.32, *p* = 1.2E-8), high beta (0.32–0.37, *p* = 1.2E-8), and low gamma coherence (0.30–0.32, *p* = 0.0042) with stimulation. Individual subject responses to stimulation are summarized in Supplementary Fig. 4. These results were further supported by mixed-effects interaction analysis, which examined whether differences in spectral features between responders and non-responders differed. Except for increased ON medication GPi-GPe coherence for medication responders, the significance of these findings was redemonstrated (Supplementary Tables 5–8).

Because our exploration of neural features associated with improved turning underscored the relevance of beta power, we examined beta bursting dynamics, particularly average burst rate (bursts/second) per turn, burst duration, and burst amplitude across all turns for the whole beta, low beta, and high beta bands. After dividing all turn trials into “Low TPI” and “High TPI” by the median, we observed a significantly higher burst rate in the high beta band among low TPI turns for M1 (0.78 vs. 0.65, *p* = 0.0003), and in the entire beta band (GP: 0.50 vs. 0.38, *p* = 0.0005; M1: 0.47 vs. 0.38, *p* = 0.0015, Fig. 4E–G). Burst duration for the entire beta band (*r* = −0.11, *p* = 5.75E-5) and low beta (*r* = −0.03, p = 6.70E-5; Fig. 4H) was negatively correlated with TPI in the pallidum. Burst amplitude was also negatively correlated with TPI for the entire beta band (*r* = −0.31, *p* = 2.96E-16), low beta (*r* = −0.28, *p* = 4.70E-55), and high beta (*r* = −0.18, *p* = 1.18E-13; Fig. 4H) in the pallidum. The inverse relationship was seen for the cortex. PMC beta burst amplitude was positively correlated with TPI (*r* = 0.24, *p* = 0.0020), as well as low beta (*r* = 0.13, *p* = 0.028).

### Machine learning models can reliably identify turns and rate quality from neural spectral features

**Fig. 5:**
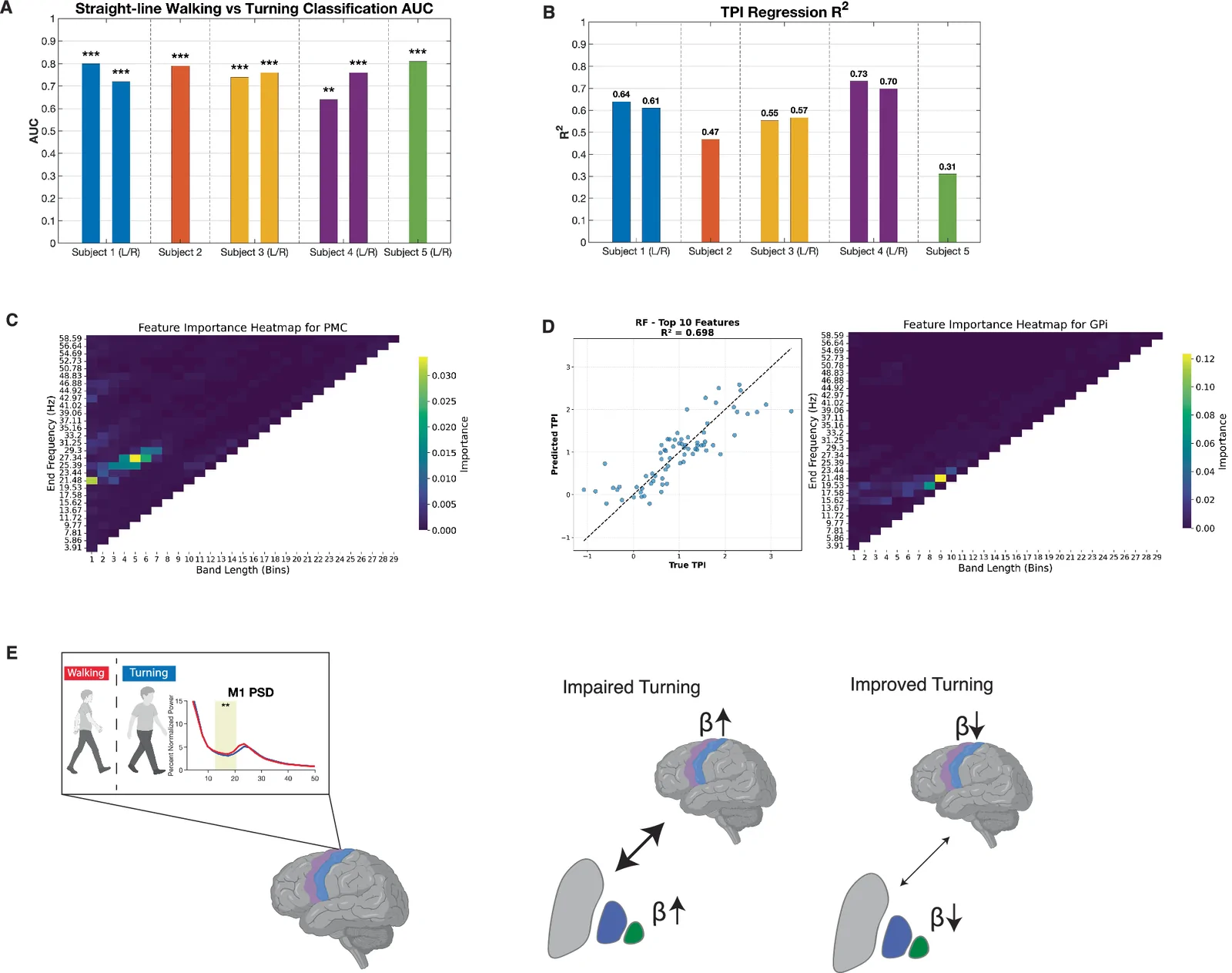
Machine learning models for labeling turns and predicting TPI. **A** Cross-validation AUC of linear discriminant analysis (LDA) classifier trained on top 20 subject specific features identified through random forest classifier for distinguishing turns from straight-line walking. **B** Cross-validation *R*^2^ values of the top performing turning performance index (TPI) prediction models for each subject. **C** Example feature importance heatmap for the primary motor cortex (PMC) for a single subject. **D** Residual plot of predicted versus true TPI along with a heatmap of regression feature importance for one subject. **E** Summary schematic showing the use of cortical beta power to separate turning from walking and the cortico-pallidal spectral changes underlying improved and impaired turning.

Finally, we sought to combine our insights from describing the neurophysiologic differences between straight-line walking and turning with our novel grading of turning performance into a machine learning approach to classify and rate turns from neural recordings alone. Because the bidirectional neural interface used in our study supports on-board classification with LDA, we trained LDA models with the top 20 subject specific features for distinguishing between straight-line walking and turning identified through a random forest classifier. Significantly better than chance AUC model performance was achieved for all five subjects, with AUC ranging from 0.64 to 0.81, and all subjects showing AUC greater than or equal to 0.7 in at least one hemisphere (Fig. 5A). Feature importance was visualized with heatmaps, with an example shown in Fig. 5B. Once again, cortical features seemed most salient for distinguishing between straight-line walking and turning.

TPI regression was performed through first ranking all subject specific features by importance, optimizing model hyperparameters among various possible models, and selecting the best performing model. The final cross validation *R*^2^ values ranged from 0.31 to 0.73 (Fig. 5C). Feature importance heatmaps for both classification and regression models, as well as residual plots for the best performing models for each subject are shown in Supplementary Fig. 5. The residual plot from subject 4 using the best performing model, a random forest regressor, as well as the feature importance heatmap from the GPi, the brain region with the greatest number of important features among the top 20 features is shown in Fig. 5D. Overall, regression analysis identified a mixture of both cortical and pallidal features as playing important roles in predicting TPI (Supplementary Fig. 5).

## Discussion

Axial motor symptoms are a uniquely disabling feature of PD due to their ubiquity and poor response to treatment. Turning, in particular, is a routine but highly demanding axial motor behavior, and its impairment is a major cause of falls and morbidity in PD. By combining invasive recordings from pallidal and cortical sites with quantitative kinematic measures, our study proposes circuit signatures that distinguish effective from impaired turning. Our results suggest that pathological beta synchronization within cortico-pallidal networks may rigidly lock the motor system into maladaptive states, whereas successful turning requires dynamic desynchronization and flexible network reorganization.

Turning was consistently associated with suppression of low- and high-beta activity in M1 and PMC, accompanied by reduced cortico-cortical and cortico-pallidal coherence (Fig. 5E), differentiating it from straight line walking. In our previous work, we demonstrated through chronic invasive cortical and basal ganglia recordings that straight-line overground walking is associated with low frequency power changes in the subthalamic nucleus and its coherence with M1 time-locked to specific phases of the gait cycle^31^. These gait-phase associated neural dynamics were also seen in the pallidum, and we can use these biomarkers to distinguish periods of contralateral leg swing during straight-line walking^32^. In contrast, turning lacked these cyclical oscillations and instead exhibited a global suppression of beta activity across the pallido-cortical network.

Although studies of neurophysiological changes during turns are sparse, we recently reported pallidal and cortical alpha-beta desynchronization during anticipatory postural adjustments in a gait initiation task in PD patients, just prior to the stepping foot leaving the ground^33^. Additionally, EEG evidence from balance perturbation experiments found that cortical alpha-beta desynchronizations are associated with balance perturbations as well as with voluntary ML and AP sway^25,34^. Together, these findings suggest that pallidal-cortical beta desynchronization may represent a neurophysiologic correlate of continuous postural transition. In our data, these dynamics correlated with pelvic angular acceleration, suggesting that cortical beta desynchronization may permit the flexibility required to reorient the body axis. By contrast, impaired turns were marked by persistent pallidal and cortical beta bursts, reflecting a temporal rigidity which may constrain adaptive control. This supports a mechanistic model in which beta synchronization enforces the motor “status quo,” while its disruption is essential for initiating and executing axial reorientation^35–41^.

Our findings also suggest that dopamine and DBS may improve turning through distinct yet complementary circuit pathways. In PD, improvements in motor symptoms with levodopa and DBS have been linked to reduced pallidal beta and cortico-pallidal synchronization in the beta band^42–44^. During simple upper extremity tasks, improved performance on levodopa corresponds to greater cortico-basal ganglia desynchronization at motor onset^45^. DBS and levodopa can also synergistically improve task performance, as they may preferentially reduce pallidal high and low beta oscillatory power, respectively^46^.

Our findings extend the current understanding of beta dynamics and their relationship with motor performance by showing turning-specific modulation in power and connectivity. Specifically, we show that reductions in low and high beta power both in the GPi and GPe as well as the cortex are associated with improved turning. Medication responders for turning exhibited broad suppression of GPi and GPe beta activity and reduced pallido-cortical coherence, consistent with dopamine restoring basal ganglia throughput. DBS responders for turning, in contrast, showed cortical beta suppression and decreased M1–PMC coherence, underscoring cortical network reorganization as a key therapeutic effect of stimulation. These complementary mechanisms suggest that pallidal and cortical beta represent parallel entry points for restoring motor flexibility during turning.

Despite the overall efficacy of DBS for appendicular motor symptoms, its impact on axial control is inconsistent^1,19,21^. In our cohort, some patients demonstrated worsened turning with stimulation, characterized by paradoxical elevations in cortical beta power and M1–PMC coherence compared to ON medication states. Rather than releasing the network from rigid synchronization, stimulation in these individuals may have reinforced maladaptive cortico-cortical coupling, constraining the dynamic flexibility required for turning.

While DBS generally improves motor functions by decreasing basal ganglia and basal ganglia-cortical synchrony, some studies report that DBS can, under certain conditions, increase cortical high beta synchronization^42,47,48^. In a study comparing resting-state cortical connectivity between DBS on and off states, the authors found that turning DBS on increased high beta and low gamma band synchrony in a cortical circuit spanning the motor, occipitoparietal, middle temporal, and prefrontal cortices^49^. Additionally, ventral GPi stimulation has been associated with bradykinesia by inhibiting the pedunculopontine nucleus, involved with gait and postural control, or suppressing the motor drive through pallidothalamic fibers^50–52^. Another possibility is that the elevations in M1 beta power and M1-PMC coherence in the DBS ON state reflect state-specific biomarkers of impaired axial control rather than direct stimulation effects.

Together, these observations underscore that the therapeutic variability of DBS on axial behaviors likely reflects a delicate balance: when stimulation successfully suppresses pathological cortical beta and reduces cortico-cortical coupling, turning may improve; when stimulation paradoxically enhances cortical beta synchronization, turning may deteriorate. This mechanistic framework suggests that cortical biomarkers may help identify responders versus non-responders and guide individualized programming to avoid maladaptive entrainment of cortical rhythms, however validation in larger cohorts is needed.

We demonstrate feasibility of using LDA classifiers, the on-board classifier for the neural interface used in our study, to identify turns from straight-line walking using neural signals alone. All subjects achieved an AUC greater than 0.7 in at least one hemisphere, and subject-specific models predicted turning performance with high accuracy. Such approaches could support real-time assessment of turning quality in naturalistic settings and inform adaptive DBS programming tailored to axial performance.

Reliable neural correlates of freezing of gait, impaired turning, and other aspects of gait dysfunction remain an unmet need in PD^53^. Accurate movement state classification and identification of symptom severity based on biomarkers is a promising strategy for adaptive DBS in PD^32,53–55^. Our integration of pallidal LFP and cortical ECoG recordings is a major strength, enabling both the identification of common network features and the training of individualized models for turn detection and grading. Beyond offline analysis, this framework could dynamically recognize turning as a distinct movement state and transiently adjust stimulation to a gait-optimized setting to improve or stabilize axial function.

This study has several limitations. Although our analysis represents a novel approach to investigating intracranial neurophysiology during turns, our sample size is limited to five subjects due to the invasive nature of this surgical study with investigational devices. The analysis of responders and non-responders should be considered exploratory given the limited statistical power. In addition, patient-specific disease factors may limit generalization to broader PD populations or to healthy controls. Although the TPI showed robust agreement with clinician ratings, validation in larger cohorts will be required to confirm external reproducibility. Furthermore, invasive recordings from larger cohorts of patients would enable us to better delineate broad, universal spectral features of impaired turning from patient specific biomarkers. Finally, because the feature importance ranking was derived from the full dataset prior to model evaluation in our machine learning analysis, our reported AUC and R² values may be optimistically biased.

Our findings are consistent with a mechanistic model in which sub-optimal turning in PD arises from pathological cortico-pallidal beta synchronization that locks the motor system into inflexible states. Effective turning depends on dynamic suppression of beta power and reduction of pathological coherence, enabling flexible reorganization of motor networks. Dopamine and DBS appear to restore turning through distinct circuit pathways, pallidal versus cortical beta suppression, revealing parallel control points for therapeutic modulation. By delineating the oscillatory and connectivity dynamics that distinguish effective from impaired turning, we establish turning as a model behavior for probing axial dysfunction and as a circuit-level biomarker for adaptive stimulation. These insights lay the groundwork for next-generation neuromodulation strategies that dynamically disrupt pathological beta synchronization to restore flexibility in human motor control.

## Methods

### Patient selection, surgical procedure, and electrode localization

Five patients with idiopathic PD (three male, two females age range 57–71, Supplementary Table 1) underwent implantation of a bidirectional neural interface (Summit RC + S, Medtronic Inc.) as part of an ongoing clinical trial (ClinicalTrials.gov ID: NCT-0358289) with IRB approval (UCSF IRB# 20-32847). All five patients gave their informed consent to participate in this trial following multiple conversations with study investigators in which the details of study enrollment, including risks related to the study device, were explained to them. The research was performed in accordance with the Declaration of Helsinki. Inclusion criteria were surgical candidacy for DBS, baseline MoCA (Montreal Cognitive Assessment) score of at least 21, and disease duration of at least four years.

The device streamed local field potentials (LFPs) from the globus pallidus internus (GPi) and externus (GPe) and recorded electrocorticography (ECoG) potentials from primary motor (M1) and premotor cortices (PMC) (Fig. 1A–C). Electrode localization followed established pipelines^31,33,53,54,56–60^. Four patients received bilateral implants; subject 5 underwent left-sided unilateral implantation. For subject 2, only left-sided data were analyzed due to a staged right implantation 10 months later.

### Experimental setup, data collection, and data alignment

Subjects performed multiple trials of an overground walking task consisting of approximately 200 non-turning steps in a 6-meter loop. At each end, patients executed 180° turns to the left (counterclockwise) or right (clockwise) as instructed before the trial start. Walking tasks were completed in the ON and OFF medication states prior to DBS activation. The ON and OFF medication (ON/OFF med) states were defined as previously described^33^. Briefly, ON med was defined as 30 min following the last dose, and OFF med as withholding one or more doses, as tolerated. Patients then performed the walking tasks after clinical DBS optimization in both medication states (if tolerated, Fig. 1D).

During all trials, surface EMG (Tirgno, Delsys Inc., Natik, MA) and inertial measurement units (IMU, Xsens, Movella, Netherlands) collected biomechanical measurements. Force-sensitive resistors (FSRs) were placed under the calcaneus, hallux, and first and fifth metatarsal heads to capture gait events. Neural data were continuously streamed at 500 Hz from GPi and GPe contact pairs (0–2, 1–3) and from adjacent ECoG electrodes over M1 and PMC. Data were processed using open-source pipelines (https://github.com/openmind-consortium/Analysis-rcs-data) as described previously^31,33,53,54,61^.

Neural, EMG, and IMU signals were aligned in MATLAB (Mathworks, Inc., Natick, MA, version 2024b) by synchronizing peaks from the on-board accelerometer, IMU, and EMG sensors, following previously described methods^31,33,53,54,62^.

#### Kinematic signal processing and turn epoch identification

All kinematic signals were sampled at 60 Hz and were low-pass filtered in MATLAB using a 1.5 Hz 4th order Butterworth filter. Turns were identified using patient-specific pelvic angular velocity thresholds of 30–70 degrees/second, as validated previously^7^. Turning epochs began and ended at the times pelvic angular velocity exceeded and fell below threshold, respectively. Straight-line walking epochs were defined as all steps outside turning epochs, excluding the stride immediately before and after each turn.

#### Definition and validation of the TPI

Kinematic parameters associated with turning performance were identified from literature search^5–11,13,28–30^. A correlation matrix of all variables is shown in Fig. 3A. Stability was defined following published descriptions^13,63–65^. Briefly, the base of support (BOS) was bounded laterally by line segments through the positions of the foot and toe as reported by Xsens and anteriorly by lines perpendicular to these segments. The lateral and anterior-posterior (AP) margins of stability (MOS) were computed as the perpendicular distances from the center of mass (COM) to the corresponding BOS borders. The inverted pendulum model was used to derive extrapolated COM (ECOM) positions:

$$X{E}_{{COM}}(t)\,={X}_{{COM}}({\rm{t}})\,+\frac{{\dot{Y}}_{{COM}}(t)}{{\omega }_{0}}$$

$$Y{E}_{{COM}}(t)\,={Y}_{{COM}}({\rm{t}})\,+\frac{{\dot{Y}}_{{COM}}(t)}{{\omega }_{0}}$$

$${\omega }_{0}=\sqrt{\frac{g}{l}}$$

Where ${\omega }_{0}$ is a constant, computed from the gravitational constant, *g* (9.81 m/s^2^), and pendulum length, *l*, which is the mean distance from the COM to the foot. From these, we calculated COM and ECOM percent stable and minimum MOS in both lateral and AP directions. Percent stable reflected the proportion of the turn during which the COM or ECOM remained within the BOS (Fig. 3D). Minimum MOS represented the smaller of left/right MOS values. For AP MOS, only the leading foot was considered.

Principal component analysis (PCA) identified kinematic variables most correlated with dominant axes of variation, guiding feature selection for the TPI. A composite stability metric was defined as the mean of the four lateral stability measures (COM and ECOM minimum MOS, COM and ECOM percent stable). The final TPI was defined as

$${TPI}={w}_{a}* {\omega }_{{pelvis}}+\,{w}_{s}* s+\,{w}_{v}* {v}_{{pelvis}}$$

where ${\omega }_{{pelvis}}$ represents pelvic angular velocity, *s* is composite stability, and ${v}_{{pelvis}}$ is linear velocity measured at the pelvis.

TPI validity was assessed by correlating TPI values with blinded physical therapist ratings of 40 randomly selected turns across five subjects (ordinal scale 1–8). In addition, two reviewers evaluated turning performance overall across conditions within each subject and assigned ordinal ranks. A third reviewer broke any ties. Model coefficients were selected by optimizing Spearman correlation such that the absolute value of coefficients sum to one. To prevent overfitting, an additional optimization constraint was the overall TPI ratings of conditions agreed with the clinical assessment. The optimal weights found were:

$${w}_{a}=0.6$$

$${w}_{s}=-0.05$$

$${w}_{v}=0.35$$

#### Neural signal processing

LFPs were high-pass filtered at 2 Hz using a Butterworth filter. Stimulation artifacts were removed by notch filtering, and EKG contamination was eliminated via template subtraction when present^66^. Channels referenced to the stimulating electrode were excluded from ON-stimulation analyses due to residual artifacts. High-frequency artifacts were detected using a custom algorithm^62^. Signals were bandpass filtered from 75 to 150 Hz, transformed using multitaper spectral estimates, Z-normalized, and trials exceeding patient-specific z-score thresholds (30–80) were excluded.

PSD (power spectral density) was calculated using Welch’s method implemented through “pwelch” in MATLAB (window size 250 samples, 90% overlap, 256 NFFT). For epochs shorter than 500 ms, the largest window size smaller than the epoch length was used. Absolute power was computed using the STFT (short-time Fourier Transform) through the “spectrogram” function with identical parameters and was also used to generate time-frequency spectrograms. Coherence was analyzed through wavelet coherence using “wcoherence” (2–150 Hz, 32 voices per octave). Analyses focused on canonical frequency bands: theta (4–8 Hz), alpha (8.5–12 Hz), low beta (13–20 Hz), high beta (20.5–30 Hz), and low gamma (30.5–50 Hz).

When comparing PSDs across multiple conditions and aggregating the results of all subjects, percent normalized power was defined as the power in each frequency bin divided by the total power from 2 to 50 Hz. Trials were normalized and pooled across hemispheres for bilaterally implanted patients.

Spearman’s correlation was conducted between kinematic variables and absolute power, normalized power, and beta burst features. For kinematic variables such as angular acceleration that were sampled throughout a turn, point-by-point correlation was performed with average power at that given time point. Otherwise, trial level turn metrics were correlated with power averaged across each trial.

Filtered neural signals were first Z-normalized and bandpass filtered for the low beta, high beta, or entire beta band. Beta-bursts longer than 100 ms in duration and exceeding the 75th percentile of the signal’s amplitude envelope were considered for downstream analysis^67–70^. Burst duration and amplitude were correlated with TPI while burst rate was dichotomized into “low” and “high” TPI groups using the median split.

Model training and assessment was performed using Python (version 3.9.21) and the scikit-learn library (version 1.1.3). LDA (linear discriminant analysis) classifiers were trained individually on subjects. First, all straight-line walking and turning epochs were divided into non-overlapping 2-s windows, with smaller windows permitted for shorter epochs. For each brain region, average power in the canonical frequency bands and in each possible frequency band from 2 to 50 Hz was extracted in addition to average coherence across regions for these bands, yielding 4401 features per subject.

A random forest classifier (*n* = 1000 trees, all variables sampled at each split) was trained using 5-fold CV (cross validation) to rank feature importance. The top 20 features were selected to train an LDA classifier using 5-fold CV on the entire training dataset. The cross-validation AUC was reported as the final AUC. To predict TPI, a random forest regressor was first trained using 5-fold CV on all features to rank features by mean importance. A model comparison was then performed across five architectures (random forest regression, gradient boosted regression, LASSO, Elastic Net, and ridge regression) and five feature subset sizes (top 5, 10, 20, 50, and 100 features from the importance ranking). Each architecture–feature count combination was evaluated using 5-fold CV, with the combination yielding the highest mean *R*² selected as the final model.

### Linear mixed-effects analysis

Linear mixed-effects models were used to account for clustering of multiple epochs within individual hemispheres. Models were implemented in R (version 4.4.2) using the lme4 (version 1.1-36) and lmerTest (version 3.2-1) packages, with *p*-values for fixed effects obtained via t-tests using Satterthwaite’s approximation for degrees of freedom.

To analyze spectral differences between straight-line walking and turning, the following models were fit for each brain region and frequency band:

$${\rm{Power}} \sim {\rm{Condition}}+(1|{\rm{Subject}}:{\rm{Hemisphere}})$$

$${\rm{Coherence}} \sim {\rm{Condition}}+(1|{\rm{Subject}}:{\rm{Hemisphere}})$$

where Condition (straight-line walking vs. turning) was the fixed effect and hemisphe re was the random intercept, accounting for repeated measurements within each subject’s implanted hemisphere. Normalized power and average wavelet coherence within canonical frequency bands served as the dependent variables.

To assess whether neural changes with medication and stimulation differed between responders and non-responders, interaction models were fit:

$${\rm{Power}} \sim {\rm{Treatment}}\times {\rm{ResponderStatus}}+(1|{\rm{Subject}}:{\rm{Hemisphere}})$$

$${\rm{Coherence}} \sim {\rm{Treatment}}\times {\rm{ResponderStatus}}+(1|{\rm{Subject}}:{\rm{Hemisphere}})$$

where Treatment refers to medication state (ON vs. OFF medication) or stimulation state (ON vs. OFF stimulation), and ResponderStatus indicates whether the subject was classified as a treatment responder or non-responder based on TPI. The interaction term tests whether the treatment-induced change in neural features differs between groups.

To assess the association between beta bursting metrics and TPI, the following models were fit:

$${\rm{TPI}} \sim {\rm{burst\; metric}}+(1|{\rm{Subject}}:{\rm{Hemisphere}})$$

where burst metric refers to burst duration or amplitude, for either the low beta, high beta, or entire beta band.

To assess the within-turn temporal relationship between pelvic angular acceleration and band power, models were fit per subject using the nlme (version 3.1-166) package in R:

$${\rm{Angular\; Acceleration}} \sim {\rm{Power}}+(1|{\rm{Turn}}),{\rm{correlation}}={\rm{corAR}}1( \sim {\rm{time}}|{\rm{Turn}})$$

The random intercept (1 | Turn) accounts for per-turn baseline differences, while the corAR1 term specifies that residuals within each turn are temporally autocorrelated, with the correlation between two time points decaying as *ρ*ᵏ where *k* is their temporal separation and *ρ* is estimated from the data. Both variables were z-scored prior to model fitting for numerical stability.

### Statistical analysis

Continuous variables were compared using two-sided Wilcoxon rank-sum tests with Benjamini-Hochberg correction, implemented using the “fdr_bh” function in MATLAB. Adjustment for multiple comparisons was applied to all comparisons made per channel, and all *p* values reported have been adjusted.

Classifier AUC significance was assessed by 1000-iteration permutation testing, comparing model AUC to the null distribution of permuted labels.

## Supplementary information

Supplementary Material

## Data Availability

The data, MATLAB, and Python scripts used in this study can be made available upon reasonable request to the corresponding author. All patient confidentiality and disclosure standards must be adhered to.

## Code availability

The MATLAB and Python scripts used in this study can be made available upon reasonable request to the corresponding author.

## References

[1] Fasano, A., Aquino, C. C., Krauss, J. K., Honey, C. R. & Bloem, B. R. Axial disability and deep brain stimulation in patients with Parkinson disease. *Nat. Rev. Neurol.* **11**, 98–110 (2015). 25582445 10.1038/nrneurol.2014.252

[2] Glaister, B. C., Bernatz, G. C., Klute, G. K. & Orendurff, M. S. Video task analysis of turning during activities of daily living. *Gait Posture* **25**, 289–294 (2007). 16730441 10.1016/j.gaitpost.2006.04.003

[3] Mancini, M. et al. Continuous monitoring of turning in Parkinson’s disease: rehabilitation potential. *NeuroRehabilitation* **37**, 3 (2015). 26409689 10.3233/NRE-151236 PMC4745985

[4] McNeely, M. E. & Earhart, G. M. The effects of medication on turning in people with Parkinson disease with and without freezing of gait. *J. Parkinsons Dis.* **1**, 259 (2011). 23939306 10.3233/JPD-2011-11030 PMC3912574

[5] Stack, E. L., Ashburn, A. M. & Jupp, K. E. Strategies used by people with Parkinson’s disease who report difficulty turning. *Parkinson. Relat. Disord.* **12**, 87–92 (2006). 10.1016/j.parkreldis.2005.08.008 16338159

[6] Adamson, M. B., Gilmore, G., Stratton, T. W., Baktash, N. & Jog, M. S. Medication status and dual-tasking on turning strategies in Parkinson disease. *J. Neurol. Sci.* **396**, 206–212 (2019). 30504066 10.1016/j.jns.2018.11.028

[7] El-Gohary, M. et al. Continuous monitoring of turning in patients with movement disability. *Sensors* **14**, 356 (2014). 10.3390/s140100356 PMC3926561 24379043

[8] Bengevoord, A. et al. Center of mass trajectories during turning in patients with Parkinson’s disease with and without freezing of gait. *Gait Posture* **43**, 54–59 (2016). 26669952 10.1016/j.gaitpost.2015.10.021

[9] Hulbert, S., Ashburn, A., Robert, L. & Verheyden, G. A narrative review of turning deficits in people with Parkinson’s disease. *Disabil. Rehabil.* **37**, 1382–1389 (2015). 25255298 10.3109/09638288.2014.961661

[10] Bertoli, M., Croce, U. D., Cereatti, A. & Mancini, M. Objective measures to investigate turning impairments and freezing of gait in people with Parkinson’s disease. *Gait Posture* **74**, 187–193 (2019). 31539800 10.1016/j.gaitpost.2019.09.001

[11] Mellone, S., Mancini, M., King, L. A., Horak, F. B. & Chiari, L. The quality of turning in Parkinson’s disease: a compensatory strategy to prevent postural instability? *J. Neuroeng. Rehabil.* **13**, 1–9 (2016). 27094039 10.1186/s12984-016-0147-4 PMC4837520

[12] Mancini, M. et al. The clinical significance of freezing while turning in Parkinson’s disease. *Neuroscience* **343**, 222 (2017). 27956066 10.1016/j.neuroscience.2016.11.045 PMC5289743

[13] Conradsson, D., Paquette, C. & Franzén, E. Turning stability in individuals with Parkinson disease. *J. Neurol. Phys. Ther.* **42**, 241–247 (2018). 30138233 10.1097/NPT.0000000000000242

[14] Schlenstedt, C. et al. The effect of medication and deep brain stimulation on posture in Parkinson’s disease. *Front. Neurol.* **10**, 499495 (2019). 10.3389/fneur.2019.01254 PMC6901659 31849818

[15] Smulders, K., Dale, M. L., Carlson-Kuhta, P., Nutt, J. G. & Horak, F. B. Pharmacological treatment in Parkinson’s disease: effects on gait. *Parkinson. Relat. Disord.* **31**, 3 (2016). 10.1016/j.parkreldis.2016.07.006 PMC5048566 27461783

[16] Curtze, C., Nutt, J. G., Carlson-Kuhta, P., Mancini, M. & Horak, F. B. Levodopa is a double-edged sword for balance and gait in people with Parkinson’s disease. *Mov. Disord.* **30**, 1361 (2015). 26095928 10.1002/mds.26269 PMC4755510

[17] Pillai, L., Landes, R. D. & Virmani, T. Levodopa influence on turning dynamics in people with Parkinson’s disease. *Sci. Rep.* **15**, 14141 (2025). 40269077 10.1038/s41598-025-97511-4 PMC12019590

[18] Conradsson, D., Paquette, C., Lökk, J. & Franzén, E. Pre- and unplanned walking turns in Parkinson’s disease – Effects of dopaminergic medication. *Neuroscience* **341**, 18–26 (2017). 27867059 10.1016/j.neuroscience.2016.11.016

[19] Collomb-Clerc, A. & Welter, M. L. Effects of deep brain stimulation on balance and gait in patients with Parkinson’s disease: a systematic neurophysiological review. *Neurophysiol. Clin.* **45**, 371–388 (2015). 26319759 10.1016/j.neucli.2015.07.001

[20] Cani, I. et al. Evaluating gait and postural responses to subthalamic stimulation and levodopa: a prospective study using wearable technology. *Eur. J. Neurol.* **32**, e16580 (2024). 10.1111/ene.16580 PMC11658965 39702816

[21] Daalen, J. M. J. et al. Gait and balance worsening after bilateral deep brain stimulation of the subthalamic nucleus (STN-DBS) for Parkinson’s disease: a systematic review. *BMJ Neurol. Open* **7**, e000898 (2025). 10.1136/bmjno-2024-000898 PMC11907047 40092840

[22] Potter, K. & Brandfass, K. The mini-balance evaluation systems test (Mini-BESTest). *J. Physiother.* **61**, 225 (2015). 26044345 10.1016/j.jphys.2015.04.002

[23] Gavriliuc, O. et al. Spin turns in advanced Parkinson’s disease: a new clinical gait sign? *Parkinson. Relat. Disord.* **69**, 19–22 (2019). 10.1016/j.parkreldis.2019.10.011 31655459

[24] Gilat, M. et al. Brain activation underlying turning in Parkinson’s disease patients with and without freezing of gait: a virtual reality fMRI study. *NPJ Parkinsons Dis.* **1**, 15020 (2015). 28725687 10.1038/npjparkd.2015.20 PMC5516618

[25] Bath, J. E. & Wang, D. D. Unraveling the threads of stability: a review of the neurophysiology of postural control in Parkinson’s disease. *Neurotherapeutics* **21**, e00354 (2024). 38579454 10.1016/j.neurot.2024.e00354 PMC11000188

[26] Takakusaki, K. Functional neuroanatomy for posture and gait control. *J. Mov. Disord.* **10**, 1 (2017). 28122432 10.14802/jmd.16062 PMC5288669

[27] Takakusaki, K., Takahashi, M., Noguchi, T. & Chiba, R. Neurophysiological mechanisms of gait disturbance in advanced Parkinson’s disease patients. *Neurol. Clin. Neurosci.* **11**, 201–217 (2023).

[28] Miri, A. L. et al. A biomechanical analysis of turning during gait in individuals with different subtypes of Parkinson’s disease. *Clin. Biomech.* **112**, 106166 (2024). 10.1016/j.clinbiomech.2023.106166 38198906

[29] Stack, E., Jupp, K. & Ashburn, A. Developing methods to evaluate how people with Parkinson’s Disease turn 180°: an activity frequently associated with falls. *Disabil. Rehabil.* **26**, 478–484 (2004). 15204470 10.1080/09638280410001663085

[30] Hase, K. & Stein, R. B. Turning strategies during human walking. *J. Neurophysiol.* **81**, 2914–2922 (1999). 10368408 10.1152/jn.1999.81.6.2914

[31] Louie K. H., et al. Disorders of the nervous system cortico-subthalamic field potentials support classification of the natural gait cycle in Parkinson’s disease and reveal individualized spectral signatures. 10.1523/ENEURO.0325-22.2022 (2022). PMC9663205 36270803

[32] Louie, K. H. et al. Adaptive deep brain stimulation timed to gait phase improves walking in Parkinson’s disease. Preprint at 10.1101/2025.08.19.25333759 (2025).

[33] Bath, J. E. et al. Pallidal and motor cortical interactions determine gait initiation dynamics in Parkinson’s disease. Running Title: neural dynamics of gait initiation. Corresponding Author. 10.1101/2024.12.12.24318797.

[34] Peterson, S. M. & Ferris, D. P. Differentiation in theta and beta electrocortical activity between visual and physical perturbations to walking and standing balance. *eNeuro* **5**, ENEURO.0207-18.2018 (2018). 30105299 10.1523/ENEURO.0207-18.2018 PMC6088363

[35] Engel, A. K. & Fries, P. Beta-band oscillations — signalling the status quo? *Curr. Opin. Neurobiol.* **20**, 156–165 (2010). 20359884 10.1016/j.conb.2010.02.015

[36] Allen, D. P. & MacKinnon, C. D. Time–frequency analysis of movement-related spectral power in EEG during repetitive movements: a comparison of methods. *J. Neurosci. Methods* **186**, 107–115 (2010). 19909774 10.1016/j.jneumeth.2009.10.022 PMC2815165

[37] Chung, J. W., Ofori, E., Misra, G., Hess, C. W. & Vaillancourt, D. E. Beta-band activity and connectivity in sensorimotor and parietal cortex are important for accurate motor performance. *Neuroimage* **144**, 164 (2016). 27746389 10.1016/j.neuroimage.2016.10.008 PMC5183516

[38] Lau, T. M., Gwin, J. T. & Ferris, D. P. Walking reduces sensorimotor network connectivity compared to standing. *J. Neuroeng. Rehabil.* **11**, 14 (2014). 24524394 10.1186/1743-0003-11-14 PMC3929753

[39] Cross, K. A., Malekmohammadi, M., Woo Choi, J. & Pouratian, N. Movement-related changes in pallidocortical synchrony differentiate action execution and observation in humans. *Clin. Neurophysiol.* **132**, 1990 (2021). 33980469 10.1016/j.clinph.2021.03.037 PMC8286311

[40] van Wijk, B. C. M. et al. Low-beta cortico-pallidal coherence decreases during movement and correlates with overall reaction time. *Neuroimage* **159**, 1–8 (2017). 28712991 10.1016/j.neuroimage.2017.07.024 PMC5678295

[41] Asher, E. E. et al. Connectivity of EEG synchronization networks increases for Parkinson’s disease patients with freezing of gait. *Commun. Biol.* **4**, 1–10 (2021). 34462540 10.1038/s42003-021-02544-w PMC8405655

[42] Wang, D. D. et al. Pallidal deep-brain stimulation disrupts pallidal beta oscillations and coherence with primary motor cortex in Parkinson’s disease. *J. Neurosci.* **38**, 4556 (2018). 29661966 10.1523/JNEUROSCI.0431-18.2018 PMC5943981

[43] Wang, D. D. et al. Subthalamic local field potentials in Parkinson’s disease and isolated dystonia: an evaluation of potential biomarkers. *Neurobiol. Dis.* **89**, 213 (2016). 26884091 10.1016/j.nbd.2016.02.015 PMC4790450

[44] Kühn, A. A. et al. High-Frequency Stimulation of the Subthalamic Nucleus Suppresses Oscillatory β Activity in Patients with Parkinson’s Disease in Parallel with Improvement in Motor Performance. *J. Neurosci.* **28**, 6165–6173 (2008). 18550758 10.1523/JNEUROSCI.0282-08.2008 PMC6670522

[45] Fischer, P. et al. Beta synchrony in the cortico-basal ganglia network during regulation of force control on and off dopamine. *Neurobiol. Dis.* **127**, 253–263 (2019). 30849510 10.1016/j.nbd.2019.03.004 PMC6517271

[46] Mathiopoulou, V. et al. Modulation of subthalamic beta oscillations by movement, dopamine, and deep brain stimulation in Parkinson’s disease. *npj Parkinsons Dis.* **10**, 1–7 (2024). 2024 10:1. 38580641 10.1038/s41531-024-00693-3 PMC10997749

[47] De Hemptinne, C. et al. Therapeutic deep brain stimulation reduces cortical phase-amplitude coupling in Parkinson’s disease. *Nat. Neurosci.* **18**, 779 (2015). 25867121 10.1038/nn.3997 PMC4414895

[48] De Hemptinne, C. et al. Exaggerated phase-amplitude coupling in the primary motor cortex in Parkinson disease. *Proc. Natl. Acad. Sci. USA* **110**, 4780–4785 (2013). 23471992 10.1073/pnas.1214546110 PMC3606991

[49] Wang, M. B., Boring, M. J., Ward, M. J., Richardson, R. M. & Ghuman, A. S. Deep brain stimulation for parkinson’s disease induces spontaneous cortical hypersynchrony in extended motor and cognitive networks. *Cereb. Cortex* **32**, 4480 (2022). 35136991 10.1093/cercor/bhab496 PMC9574237

[50] Kumar, R. et al. Deep brain stimulation of the globus pallidus pars interna in advanced Parkinson’s disease. *Neurology* **55**, S34–S39 (2000). 11188973

[51] Mahlknecht, P. et al. Performance of the movement disorders society criteria for prodromal Parkinson’s disease: a population-based 10-year study. *Mov. Disord.* **33**, 405–413 (2018). 29436728 10.1002/mds.27281

[52] Galazky, I. et al. Pallidal stimulation modulates pedunculopontine nuclei in Parkinson’s disease. *Brain Sci.* **8**, 117 (2018). 29941788 10.3390/brainsci8070117 PMC6071240

[53] Fekri Azgomi, H. et al. Modeling and optimizing deep brain stimulation to enhance gait in Parkinson’s disease: personalized treatment with neurophysiological insights. *npj Parkinsons Dis.* **11**, 1–16 (2025). 2025 11:1. 40533464 10.1038/s41531-025-00990-5 PMC12177069

[54] Ramesh, R. et al. At-home movement state classification using totally implantable cortical-basal ganglia neural interface. *Sci. Adv.* **12**, eadz4733 (2026). 41686905 10.1126/sciadv.adz4733 PMC12904197

[55] Oehrn, C. R. et al. Chronic adaptive deep brain stimulation versus conventional stimulation in Parkinson’s disease: a blinded randomized feasibility trial. *Nat. Med.* **30**, 3345–3356 (2024). 39160351 10.1038/s41591-024-03196-z PMC11826929

[56] Horn, A. & Kühn, A. A. Lead-DBS: a toolbox for deep brain stimulation electrode localizations and visualizations. *Neuroimage* **107**, 127–135 (2015). 25498389 10.1016/j.neuroimage.2014.12.002

[57] Ewert, S. et al. Toward defining deep brain stimulation targets in MNI space: a subcortical atlas based on multimodal MRI, histology and structural connectivity. *Neuroimage* **170**, 271–282 (2018). 28536045 10.1016/j.neuroimage.2017.05.015

[58] Horn, A. et al. Lead-DBS v2: towards a comprehensive pipeline for deep brain stimulation imaging. *Neuroimage* **184**, 293 (2018). 30179717 10.1016/j.neuroimage.2018.08.068 PMC6286150

[59] Davis, T. S. et al. LeGUI: a fast and accurate graphical user interface for automated detection and anatomical localization of intracranial electrodes. *Front. Neurosci.* **15**, 769872 (2021). 34955721 10.3389/fnins.2021.769872 PMC8695687

[60] Gilron, R. et al. Long-term wireless streaming of neural recordings for circuit discovery and adaptive stimulation in patients with Parkinson’s disease. *Nat. Biotechnol.* **39**, 1078 (2021). 33941932 10.1038/s41587-021-00897-5 PMC8434942

[61] Sellers, K. K. et al. Analysis-rcs-data: open-source toolbox for the ingestion, time-alignment, and visualization of sense and stimulation data from the medtronic summit RC+S System. *Front Hum. Neurosci.* **15**, 714256 (2021). 34322004 10.3389/fnhum.2021.714256 PMC8312257

[62] Shukla, P. D. et al. Human cervical epidural spinal electrogram topographically maps distinct volitional movements. Published online 2024. 10.1523/JNEUROSCI.2258-23.2024 PMC11308355 38960719

[63] McAndrew Young, P. M., Wilken, J. M. & Dingwell, J. B. Dynamic margins of stability during human walking in destabilizing environments. *J. Biomech.* **45**, 1053 (2012). 22326059 10.1016/j.jbiomech.2011.12.027 PMC3321251

[64] Conradsson, D., Paquette, C. & Franzén, E. Medio-lateral stability during walking turns in older adults. *PLoS ONE* **13**, e0198455 (2018). 29870557 10.1371/journal.pone.0198455 PMC5988272

[65] He, C. et al. Dynamic stability and spatiotemporal parameters during turning in healthy young adults. *Biomed. Eng. Online* **17**, 127 (2018). 30241535 10.1186/s12938-018-0558-5 PMC6151057

[66] Hammer, L. H., Kochanski, R. B., Starr, P. A. & Little, S. Artifact characterization and a multipurpose template-based offline removal solution for a sensing-enabled deep brain stimulation device. *Stereotact. Funct. Neurosurg.* **100**, 168 (2022). 35130555 10.1159/000521431 PMC9064887

[67] Tinkhauser, G. et al. Beta burst dynamics in Parkinson’s disease OFF and ON dopaminergic medication. *Brain* **140**, 2968 (2017). 29053865 10.1093/brain/awx252 PMC5667742

[68] Torrecillos, F. et al. Modulation of beta bursts in the subthalamic nucleus predicts motor performance. *J. Neurosci.* **38**, 8905 (2018). 30181135 10.1523/JNEUROSCI.1314-18.2018 PMC6181312

[69] Shin H., Law R., Tsutsui S., Moore C. I., Jones S. R. The rate of transient beta frequency events predicts behavior across tasks and species. *elife* **6**. 10.7554/ELIFE.29086 (2017) PMC5683757 29106374

[70] Mirpour, K. & Pouratian, N. Interaction of motor behaviour, cortical oscillations and deep brain stimulation in Parkinson’s disease. *Brain* **148**, 886–895 (2025). 39300838 10.1093/brain/awae300 PMC11884658

