# Supplementary material for: Cortico-pallidal beta dynamics underlie impaired turning in Parkinson’s disease
Source: NPJ Parkinsons Dis. 2026 Jun 6;12:219. doi: 10.1038/s41531-026-01421-9 (PMC13575165; doi:10.1038/s41531-026-01421-9)
Supplement: Supplementary file 1 [file 41531_2026_1421_MOESM1_ESM.docx]

**Supplementary Material**

| **Supplementary Table 1. Subject Demographic and Clinical Characteristics** | | | | | | |  |
| --- | --- | --- | --- | --- | --- | --- | --- |
| **Subject** | **Age (5-Year Range)** | **Gender** | **Disease Duration** | **LEDD** | **MDS-UPDRS III OFF MED (PIGD Subscore^1^)** | **MDS-UPDRS III ON MED (PIGD Subscore^1^)** | |
| 1 | 65-70 | Male | 21 | 2590 | 42 (7) | 25 (5)^*^ | |
| 2 | 60-65 | Male | 11 | 1514 | 42 (10)^*^ | 19 (3)^*^ | |
| 3 | 57-62 | Female | 8 | 1099 | 57 (3)^*^ | 29 (1)^*^ | |
| 4 | 64-69 | Male | 7 | 1025 | 31 (3)^*^ | 17 (2)^*^ | |
| 5 | 65-70 | Female | 3 | 618 | 21 (2)^*^ | 9 (2)^*^ | |

^1^PIGD Subscore calculated as the sum of MDS-UPDRS III items arising from chair (3.9), gait (3.10), freezing (3.11), postural stability (3.12), posture (3.13)

**Supplementary Table 1 Subject Demographic and Clinical Characteristics** Demographic and clinical characteristics of included subjects. Age and disease duration are calculated based on date of last trial used for analysis. LEDD (levodopa equivalent daily dose) obtained from latest preoperative data point. MDS-UPRDRS III (Movement Disorders Society-Unified Parkinson’s Disease Rating Scale) and PIGD (postural instability gait disability) as determined by a movement disorders neurologist. *Denotes virtual MDS-UPDRS III, so rigidity and pull-test not evaluated.

| **Supplementary Table 2. Turning Trials by Subject and Condition** | | | | | |  |
| --- | --- | --- | --- | --- | --- | --- |
|  | **Subject 1 (L+R)** | **Subject 2 (L only)** | **Subject 3 (L+R)** | **Subject 4 (L+R)** | **Subject 5 (L only)** | |
| Total Turns | 218 | 77 | 172 | 182 | 113 | |
| OFF Med OFF Stim | 50 | 16 | 46 | 52 | 26 | |
| ON Med OFF Stim | 54 | 29 | 42 | 56 | 26 | |
| OFF Med ON Stim | 0 | 0 | 34 | 0 | 30 | |
| ON Med ON Stim | 114 | 32 | 50 | 74 | 31 | |
| Rejected GPi | 9 (4.13%) | 7 (9.09%) | 2 (1.16%) | 31 (17.03%) | 0 (0%) | |
| Rejected GPe | 2 (0.92%) | 8 (10.39%) | 0 (0%) | 27 (14.84%) | 19 (16.81%) | |
| Rejected M1 | 7 (3.21%) | 4 (5.19%) | 0 (0%) | 24 (13.19%) | 0 (0%) | |
| Rejected PMC | 2 (0.92 %) | 8 (10.39%) | 0 (0%) | 20 (10.99%) | 0 (0%) | |

**Supplementary Table 2 Turning Trials by Subject and Condition** Total number of turning trials included available for each subject by condition. Trials in patients with intracranial recordings from both left and right hemispheres are counted separately. The number and percentage of rejected trials are also listed.

**Supplementary Table 3. Turning vs Straight-Line Walking Power**

| **Region** | **Band** | **Mean OG** | **Mean Turn** | **β** | **SE** | **p (FDR)** |  |
| --- | --- | --- | --- | --- | --- | --- | --- |
| GPi | Theta | 13.34 | 12.52 | -0.829 | 0.315 | 0.022 | * |
| GPi | Alpha | 5.96 | 6.13 | 0.152 | 0.160 | 0.570 |  |
| GPi | Low Beta | 4.39 | 4.52 | 0.150 | 0.222 | 0.622 |  |
| GPi | High Beta | 2.11 | 1.93 | -0.175 | 0.064 | 0.022 | * |
| GPi | Low Gamma | 0.80 | 0.80 | -0.002 | 0.022 | 0.935 |  |
| GPe | Theta | 12.98 | 12.73 | -0.253 | 0.241 | 0.492 |  |
| GPe | Alpha | 5.90 | 5.85 | -0.060 | 0.159 | 0.884 |  |
| GPe | Low Beta | 3.29 | 3.29 | 0.014 | 0.109 | 0.896 |  |
| GPe | High Beta | 1.97 | 1.77 | -0.196 | 0.051 | 6.95e-04 | *** |
| GPe | Low Gamma | 1.06 | 1.00 | -0.069 | 0.030 | 0.058 |  |
| M1 | Theta | 9.58 | 9.95 | 0.374 | 0.241 | 0.151 |  |
| M1 | Alpha | 4.73 | 4.70 | -0.033 | 0.123 | 0.786 |  |
| M1 | Low Beta | 3.77 | 3.37 | -0.392 | 0.104 | 8.85e-04 | *** |
| M1 | High Beta | 4.58 | 4.35 | -0.216 | 0.123 | 0.131 |  |
| M1 | Low Gamma | 1.42 | 1.51 | 0.078 | 0.032 | 0.037 | * |
| PMC | Theta | 11.34 | 11.32 | -0.003 | 0.230 | 0.990 |  |
| PMC | Alpha | 5.99 | 5.95 | -0.040 | 0.164 | 0.990 |  |
| PMC | Low Beta | 4.65 | 4.32 | -0.342 | 0.122 | 0.0085 | ** |
| PMC | High Beta | 3.93 | 3.64 | -0.286 | 0.084 | 0.0018 | ** |
| PMC | Low Gamma | 1.10 | 1.25 | 0.150 | 0.030 | 3.12e-06 | *** |

**Supplementary Table 3 Turning vs Straight-Line Walking Power** Results of linear-mixed effects modeling for power in turning vs straight-line walking trials. The model Power ~ Condition + (1 | Subject:Hemisphere) was used.

**Supplementary Table 4 Turning vs Straight Walking Coherence**

| **Region** | **Band** | **Mean OG** | **Mean Turn** | **β** | **SE** | **p (FDR)** |  |
| --- | --- | --- | --- | --- | --- | --- | --- |
| GPi-GPe | Theta | 0.36 | 0.37 | 0.005 | 0.007 | 0.619 |  |
| GPi-GPe | Alpha | 0.39 | 0.41 | 0.016 | 0.007 | 0.056 |  |
| GPi-GPe | Low Beta | 0.36 | 0.39 | 0.023 | 0.006 | 0.0023 | ** |
| GPi-GPe | High Beta | 0.34 | 0.34 | 0.001 | 0.005 | 0.815 |  |
| GPi-GPe | Low Gamma | 0.30 | 0.29 | -0.002 | 0.003 | 0.619 |  |
| GPi-M1 | Theta | 0.27 | 0.26 | -0.007 | 0.006 | 0.301 |  |
| GPi-M1 | Alpha | 0.26 | 0.25 | -0.008 | 0.004 | 0.160 |  |
| GPi-M1 | Low Beta | 0.26 | 0.26 | -0.001 | 0.004 | 0.915 |  |
| GPi-M1 | High Beta | 0.28 | 0.27 | -0.007 | 0.004 | 0.160 |  |
| GPi-M1 | Low Gamma | 0.26 | 0.27 | 0.000 | 0.002 | 0.915 |  |
| GPi-PMC | Theta | 0.27 | 0.26 | -0.001 | 0.005 | 0.807 |  |
| GPi-PMC | Alpha | 0.25 | 0.25 | -0.009 | 0.004 | 0.120 |  |
| GPi-PMC | Low Beta | 0.25 | 0.25 | -0.001 | 0.004 | 0.807 |  |
| GPi-PMC | High Beta | 0.28 | 0.26 | -0.017 | 0.004 | 7.89e-06 | *** |
| GPi-PMC | Low Gamma | 0.27 | 0.26 | -0.002 | 0.002 | 0.807 |  |
| GPe-M1 | Theta | 0.26 | 0.26 | 0.003 | 0.006 | 0.824 |  |
| GPe-M1 | Alpha | 0.25 | 0.26 | 0.004 | 0.004 | 0.824 |  |
| GPe-M1 | Low Beta | 0.26 | 0.25 | -0.002 | 0.004 | 0.824 |  |
| GPe-M1 | High Beta | 0.28 | 0.28 | -0.000 | 0.003 | 0.991 |  |
| GPe-M1 | Low Gamma | 0.26 | 0.27 | 0.006 | 0.003 | 0.064 |  |
| GPe-PMC | Theta | 0.26 | 0.26 | 0.000 | 0.005 | 0.965 |  |
| GPe-PMC | Alpha | 0.25 | 0.26 | 0.006 | 0.004 | 0.459 |  |
| GPe-PMC | Low Beta | 0.25 | 0.25 | 0.005 | 0.003 | 0.459 |  |
| GPe-PMC | High Beta | 0.27 | 0.27 | -0.002 | 0.003 | 0.836 |  |
| GPe-PMC | Low Gamma | 0.26 | 0.26 | 0.001 | 0.002 | 0.836 |  |
| M1-PMC | Theta | 0.28 | 0.27 | -0.006 | 0.006 | 0.328 |  |
| M1-PMC | Alpha | 0.27 | 0.26 | -0.012 | 0.005 | 0.013 | * |
| M1-PMC | Low Beta | 0.31 | 0.28 | -0.024 | 0.004 | 5.29e-08 | *** |
| M1-PMC | High Beta | 0.36 | 0.33 | -0.035 | 0.005 | 4.03e-13 | *** |
| M1-PMC | Low Gamma | 0.32 | 0.31 | -0.013 | 0.003 | 3.78e-05 | *** |

**Supplementary Table 4 Turning vs Straight-Line Walking Coherence** Results of linear-mixed effects modeling for coherence in turning vs straight-line walking trials. The model Coherence ~ Condition + (1 | Subject:Hemisphere) was used.

**Supplementary Table 5 Medication Responder Interaction Power**

| **Region** | **Band** | **Resp OFF** | **Resp ON** | **NonR OFF** | **NonR ON** | **Int. β** | **Int. SE** | **p (FDR)** |  |
| --- | --- | --- | --- | --- | --- | --- | --- | --- | --- |
| GPi | Theta | 9.92 | 15.13 | 12.13 | 12.04 | 5.391 | 0.749 | 8.69e-12 | *** |
| GPi | Alpha | 5.09 | 5.98 | 6.94 | 7.26 | 0.493 | 0.496 | 0.321 |  |
| GPi | Low Beta | 7.79 | 2.64 | 3.60 | 3.80 | -5.072 | 0.533 | 1.24e-18 | *** |
| GPi | High Beta | 1.80 | 1.43 | 2.55 | 2.49 | -0.368 | 0.169 | 0.037 | * |
| GPi | Low Gamma | 0.73 | 0.59 | 1.00 | 1.13 | -0.320 | 0.065 | 2.05e-06 | *** |
| GPe | Theta | 11.81 | 13.88 | 12.81 | 11.94 | 3.235 | 0.722 | 1.61e-05 | *** |
| GPe | Alpha | 5.20 | 5.72 | 6.02 | 7.04 | -0.588 | 0.484 | 0.225 |  |
| GPe | Low Beta | 4.16 | 2.33 | 3.30 | 3.69 | -2.288 | 0.281 | 2.67e-14 | *** |
| GPe | High Beta | 1.98 | 1.35 | 1.93 | 2.09 | -0.831 | 0.130 | 9.99e-10 | *** |
| GPe | Low Gamma | 0.86 | 0.93 | 1.11 | 1.24 | -0.119 | 0.084 | 0.200 |  |
| M1 | Theta | 9.26 | 9.97 | 10.40 | 10.60 | 0.947 | 0.750 | 0.346 |  |
| M1 | Alpha | 4.43 | 4.67 | 4.95 | 4.95 | 0.350 | 0.364 | 0.370 |  |
| M1 | Low Beta | 3.73 | 3.57 | 2.85 | 2.92 | -0.256 | 0.284 | 0.370 |  |
| M1 | High Beta | 4.99 | 4.02 | 3.87 | 4.44 | -1.584 | 0.357 | 6.08e-05 | *** |
| M1 | Low Gamma | 1.50 | 1.29 | 1.76 | 1.71 | -0.290 | 0.096 | 0.0065 | ** |
| PMC | Theta | 10.65 | 9.77 | 14.30 | 12.47 | 1.860 | 0.727 | 0.027 | * |
| PMC | Alpha | 5.99 | 6.05 | 5.25 | 6.41 | -0.827 | 0.499 | 0.123 |  |
| PMC | Low Beta | 4.48 | 4.64 | 3.66 | 4.09 | -0.410 | 0.357 | 0.252 |  |
| PMC | High Beta | 4.04 | 3.90 | 2.83 | 3.28 | -0.810 | 0.230 | 0.0024 | ** |
| PMC | Low Gamma | 1.23 | 1.39 | 1.07 | 1.22 | -0.182 | 0.093 | 0.085 |  |

**Supplementary Table 5 Medication Responder Interaction Power** Results of linear-mixed effects modeling for power among medication responders and non-responders. The model Power ~ Meds * ResponderStatus + (1 | Subject:Hemisphere) was used.

**Supplementary Table 6 Medication Responder Interaction Coherence**

| **Region** | **Band** | **Resp OFF** | **Resp ON** | **NonR OFF** | **NonR ON** | **Int. β** | **Int. SE** | **p (FDR)** |  |
| --- | --- | --- | --- | --- | --- | --- | --- | --- | --- |
| GPi-GPe | Theta | 0.37 | 0.35 | 0.39 | 0.38 | -0.016 | 0.024 | 0.700 |  |
| GPi-GPe | Alpha | 0.38 | 0.39 | 0.45 | 0.46 | 0.000 | 0.022 | 0.986 |  |
| GPi-GPe | Low Beta | 0.36 | 0.38 | 0.41 | 0.40 | 0.022 | 0.021 | 0.700 |  |
| GPi-GPe | High Beta | 0.32 | 0.36 | 0.34 | 0.34 | 0.033 | 0.016 | 0.201 |  |
| GPi-GPe | Low Gamma | 0.29 | 0.30 | 0.29 | 0.29 | 0.005 | 0.008 | 0.700 |  |
| GPi-M1 | Theta | 0.26 | 0.28 | 0.25 | 0.25 | 0.022 | 0.016 | 0.211 |  |
| GPi-M1 | Alpha | 0.23 | 0.25 | 0.25 | 0.26 | 0.018 | 0.013 | 0.211 |  |
| GPi-M1 | Low Beta | 0.26 | 0.26 | 0.24 | 0.25 | -0.011 | 0.011 | 0.298 |  |
| GPi-M1 | High Beta | 0.26 | 0.29 | 0.27 | 0.28 | 0.024 | 0.010 | 0.099 |  |
| GPi-M1 | Low Gamma | 0.27 | 0.26 | 0.26 | 0.27 | -0.015 | 0.007 | 0.102 |  |
| GPi-PMC | Theta | 0.26 | 0.26 | 0.26 | 0.27 | -0.005 | 0.015 | 0.735 |  |
| GPi-PMC | Alpha | 0.24 | 0.24 | 0.25 | 0.26 | -0.008 | 0.013 | 0.645 |  |
| GPi-PMC | Low Beta | 0.25 | 0.25 | 0.26 | 0.25 | 0.013 | 0.010 | 0.328 |  |
| GPi-PMC | High Beta | 0.25 | 0.27 | 0.27 | 0.26 | 0.027 | 0.010 | 0.044 | * |
| GPi-PMC | Low Gamma | 0.27 | 0.26 | 0.26 | 0.26 | -0.010 | 0.007 | 0.328 |  |
| GPe-M1 | Theta | 0.26 | 0.28 | 0.25 | 0.25 | 0.018 | 0.018 | 0.391 |  |
| GPe-M1 | Alpha | 0.25 | 0.27 | 0.25 | 0.26 | 0.006 | 0.013 | 0.628 |  |
| GPe-M1 | Low Beta | 0.27 | 0.25 | 0.24 | 0.24 | -0.017 | 0.010 | 0.166 |  |
| GPe-M1 | High Beta | 0.30 | 0.27 | 0.27 | 0.26 | -0.028 | 0.009 | 0.0059 | ** |
| GPe-M1 | Low Gamma | 0.28 | 0.26 | 0.26 | 0.26 | -0.034 | 0.007 | 1.67e-05 | *** |
| GPe-PMC | Theta | 0.27 | 0.26 | 0.27 | 0.26 | 0.003 | 0.016 | 0.873 |  |
| GPe-PMC | Alpha | 0.26 | 0.25 | 0.26 | 0.25 | 0.007 | 0.014 | 0.773 |  |
| GPe-PMC | Low Beta | 0.25 | 0.25 | 0.25 | 0.25 | -0.006 | 0.010 | 0.773 |  |
| GPe-PMC | High Beta | 0.28 | 0.27 | 0.26 | 0.27 | -0.019 | 0.009 | 0.088 |  |
| GPe-PMC | Low Gamma | 0.27 | 0.26 | 0.25 | 0.25 | -0.014 | 0.007 | 0.088 |  |
| M1-PMC | Theta | 0.27 | 0.27 | 0.28 | 0.28 | 0.016 | 0.018 | 0.455 |  |
| M1-PMC | Alpha | 0.25 | 0.27 | 0.25 | 0.26 | 0.010 | 0.014 | 0.455 |  |
| M1-PMC | Low Beta | 0.29 | 0.30 | 0.26 | 0.26 | 0.017 | 0.012 | 0.280 |  |
| M1-PMC | High Beta | 0.34 | 0.33 | 0.31 | 0.32 | -0.025 | 0.013 | 0.121 |  |
| M1-PMC | Low Gamma | 0.32 | 0.30 | 0.31 | 0.31 | -0.018 | 0.009 | 0.121 |  |

**Supplementary Table 6 Medication Responder Interaction Coherence** Results of linear-mixed effects modeling for coherence among medication responders and non-responders. The model Coherence ~ Meds * ResponderStatus + (1 | Subject:Hemisphere) was used.

**Supplementary Table 7 DBS Responder Interaction Power**

| **Region** | **Band** | **Resp OFF** | **Resp ON** | **NonR OFF** | **NonR ON** | **Int. β** | **Int. SE** | **p (FDR)** |  |
| --- | --- | --- | --- | --- | --- | --- | --- | --- | --- |
| GP | Theta | 13.61 | 15.15 | 13.28 | 13.15 | 1.376 | 1.016 | 0.221 |  |
| GP | Alpha | 7.46 | 8.71 | 6.39 | 6.71 | 0.933 | 0.651 | 0.221 |  |
| GP | Low Beta | 3.30 | 3.59 | 3.23 | 3.87 | -0.279 | 0.326 | 0.392 |  |
| GP | High Beta | 3.09 | 2.29 | 1.95 | 2.14 | -0.987 | 0.221 | 6.15e-05 | *** |
| GP | Low Gamma | 0.60 | 0.59 | 0.98 | 0.83 | 0.183 | 0.074 | 0.036 | * |
| M1 | Theta | 7.97 | 8.05 | 11.01 | 9.59 | 1.575 | 0.745 | 0.035 | * |
| M1 | Alpha | 4.09 | 4.29 | 5.01 | 6.28 | -1.109 | 0.427 | 0.012 | * |
| M1 | Low Beta | 3.69 | 3.66 | 3.23 | 4.31 | -1.242 | 0.334 | 3.78e-04 | *** |
| M1 | High Beta | 5.65 | 3.83 | 3.61 | 4.51 | -2.860 | 0.344 | 4.94e-15 | *** |
| M1 | Low Gamma | 1.86 | 2.44 | 1.27 | 1.34 | 0.584 | 0.085 | 5.77e-11 | *** |
| PMC | Theta | 8.33 | 8.66 | 11.52 | 12.73 | -1.068 | 0.717 | 0.228 |  |
| PMC | Alpha | 5.05 | 4.98 | 6.58 | 6.81 | -0.147 | 0.539 | 0.785 |  |
| PMC | Low Beta | 5.17 | 5.25 | 4.19 | 4.59 | -0.196 | 0.388 | 0.767 |  |
| PMC | High Beta | 4.64 | 4.03 | 3.35 | 3.23 | -0.724 | 0.237 | 0.0060 | ** |
| PMC | Low Gamma | 1.91 | 2.03 | 1.12 | 0.94 | 0.376 | 0.095 | 4.08e-04 | *** |

**Supplementary Table 7 DBS Responder Interaction Power** Results of linear-mixed effects modeling for power among DBS responders and non-responders. The model Power ~ Stim * ResponderStatus + (1 | Subject:Hemisphere) was used.

**Supplementary Table 8 DBS Responder Interaction Coherence**

| **Region** | **Band** | **Resp OFF** | **Resp ON** | **NonR OFF** | **NonR ON** | **Int. β** | **Int. SE** | **p (FDR)** |  |
| --- | --- | --- | --- | --- | --- | --- | --- | --- | --- |
| GP-M1 | Theta | 0.25 | 0.26 | 0.27 | 0.25 | 0.019 | 0.020 | 0.424 |  |
| GP-M1 | Alpha | 0.26 | 0.25 | 0.25 | 0.25 | -0.019 | 0.018 | 0.424 |  |
| GP-M1 | Low Beta | 0.25 | 0.24 | 0.26 | 0.26 | -0.016 | 0.014 | 0.424 |  |
| GP-M1 | High Beta | 0.31 | 0.28 | 0.29 | 0.29 | -0.037 | 0.013 | 0.023 | * |
| GP-M1 | Low Gamma | 0.26 | 0.25 | 0.27 | 0.26 | -0.005 | 0.009 | 0.594 |  |
| GP-PMC | Theta | 0.26 | 0.25 | 0.27 | 0.26 | 0.000 | 0.021 | 0.992 |  |
| GP-PMC | Alpha | 0.26 | 0.28 | 0.25 | 0.25 | 0.013 | 0.018 | 0.716 |  |
| GP-PMC | Low Beta | 0.24 | 0.25 | 0.25 | 0.25 | 0.007 | 0.012 | 0.716 |  |
| GP-PMC | High Beta | 0.29 | 0.28 | 0.26 | 0.27 | -0.013 | 0.013 | 0.716 |  |
| GP-PMC | Low Gamma | 0.25 | 0.26 | 0.26 | 0.26 | 0.011 | 0.009 | 0.716 |  |
| M1-PMC | Theta | 0.28 | 0.32 | 0.27 | 0.29 | 0.017 | 0.018 | 0.410 |  |
| M1-PMC | Alpha | 0.26 | 0.29 | 0.27 | 0.29 | 0.012 | 0.015 | 0.410 |  |
| M1-PMC | Low Beta | 0.31 | 0.30 | 0.28 | 0.32 | -0.054 | 0.012 | 3.37e-05 | *** |
| M1-PMC | High Beta | 0.35 | 0.32 | 0.32 | 0.36 | -0.089 | 0.013 | 1.10e-10 | *** |
| M1-PMC | Low Gamma | 0.31 | 0.29 | 0.30 | 0.32 | -0.032 | 0.008 | 3.40e-04 | *** |

**Supplementary Table 8 DBS Responder Interaction Coherence** Results of linear-mixed effects modeling for coherence among DBS responders and non-responders. The model Coherence ~ Stim * ResponderStatus + (1 | Subject:Hemisphere) was used.

| **Supplementary Table 9. Freezing of Gait Characterization** | | | |
| --- | --- | --- | --- |
| **Subject** | **History of FoG** | **History of FoG with Falls** | **FoG on Exam** |
| Subject 1, Pre-programming | Yes | Yes | No |
| Subject 1, ON stimulation | Yes | No | No |
| Subject 2, Pre-programming | Yes | No | Yes |
| Subject 2, ON stimulation | Yes | No | No |
| Subject 3, Pre-programming | Yes | No | No |
| Subject 3, ON stimulation | Yes | No | No |
| Subject 4, Pre-programming | Yes | No | No |
| Subject 4, ON stimulation | Yes | No | No |
| Subject 5, Pre-programming | No | No | No |
| Subject 5, ON stimulation | No | No | No |

**Supplementary Table 9 Freezing of Gait Characterization** History and physical exam findings of freezing of gait during clinic visits around the time of data collection trials. All patients underwent a pre-programming data collection visit (prior to onset of DBS stimulation) and another visit after optimal clinical stimulation parameters were found.

**Supplementary Figure 1 Individual subject neurophysiology of straight-line walking versus turning** Power spectral density **(**PSD) and inter-regional coherence changes in straight-line walking shown individually for all subjects. For all panels, * indicates *p* <0.05, ** indicates *p*<0.01, and *** indicates *p*<0.001. Shaded colored regions in spectral power and coherence plots indicate a significant difference in the specified frequency band (theta 4-8 Hz, alpha 8.5-12 Hz, low beta 13-20 Hz, high beta 20.5-30 Hz, and low gamma 30.5-50 Hz).

**Supplementary Figure 2 Pelvic angular acceleration correlation with cortical power** Heatmaps showing correlation coefficients (top row) and FDR adjusted -log10 p-values (bottom row) derived from linear-mixed effects models accounting for temporal autocorrelation for pelvic angular acceleration during all points sampled during turns versus average power in canonical bands for primary motor (M1) and premotor (PMC) cortices. Individual subject, hemisphere, and medication state results are shown in columns of heatmaps.

**Supplementary Figure 3 Individual subject neurophysiology of turning response to medication** Power spectral density **(**PSD) and inter-regional coherence changes during turns in the OFF medication state compared to the ON medication state shown for all subjects. For all panels, * indicates *p* <0.05, ** indicates *p*<0.01, and *** indicates *p*<0.001. Shaded colored regions in spectral power and coherence plots indicate a significant difference in the specified frequency band (theta 4-8 Hz, alpha 8.5-12 Hz, low beta 13-20 Hz, high beta 20.5-30 Hz, and low gamma 30.5-50 Hz).

**Supplementary Figure 4 Individual subject neurophysiology of turning response to stimulation** Power spectral density (PSD) and inter-regional coherence changes during turns in the ON stimulation and ON medication state compared to the OFF stimulation and ON medication state shown for all subjects. For all panels, * indicates *p* <0.05, ** indicates *p*<0.01, and *** indicates *p*<0.001. Shaded colored regions in spectral power and coherence plots indicate a significant difference in the specified frequency band (theta 4-8 Hz, alpha 8.5-12 Hz, low beta 13-20 Hz, high beta 20.5-30 Hz, and low gamma 30.5-50 Hz).

**Supplementary Figure 5 Feature importance and model performance of machine learning models by subject** Feature importance heatmaps from the most important brain regions for the straight-line walking versus turning classification task are shown in the first columns. In the second column, residual plots of the best performing turning performance index (TPI) regression models. Finally, feature importance heatmaps from the most important brain regions for TPI regression.

**Supplementary Figure 6 Cortical Power Correlation with Other Kinematic Variables** Heatmaps showing correlation coefficients (top row) and FDR adjusted -log10 p-values (bottom row) derived from linear-mixed effects models accounting for temporal autocorrelation for pelvic angular velocity, contralateral foot angular velocity, and contralateral foot speed during all points sampled during turns versus average power in canonical bands for primary motor (M1) and premotor (PMC) cortices. The contralateral foot is defined as the outer foot during a turn (i.e. the left foot for turning to the right and the right foot for turning to the left). Individual subject, hemisphere, and medication state results are shown in columns of heatmaps.
